# Physico-Chemical, Nutritional, and Sensory Evaluation of Two New Commercial Tomato Hybrids and Their Parental Lines

**DOI:** 10.3390/plants10112480

**Published:** 2021-11-16

**Authors:** Zoltán Felföldi, Floricuta Ranga, Sonia Ancuta Socaci, Anca Farcas, Mariola Plazas, Adriana F. Sestras, Dan Cristian Vodnar, Jaime Prohens, Radu E. Sestras

**Affiliations:** 1 Faculty of Horticulture, University of Agricultural Sciences and Veterinary Medicine of Cluj-Napoca, 3-5 Manastur St., 400372 Cluj-Napoca, Romania; zoltan.felfoldi@usamvcluj.ro (Z.F.); adriana.sestras@usamvcluj.ro (A.F.S.); 2 Private Research Station Agrosel, 268 Laminoriștilor St., 400500 Câmpia Turzii, Romania; 3 Department of Food Science and Technology, University of Agricultural Sciences and Veterinary Medicine of Cluj-Napoca, 3-5 Manastur St., 400372 Cluj-Napoca, Romania; floricuta.ranga@usamvcluj.ro (F.R.); dan.vodnar@usamvcluj.ro (D.C.V.); 4 Department of Chemistry and Biochemistry, University of Agricultural Sciences and Veterinary Medicine of Cluj-Napoca, 3-5 Manastur St., 400372 Cluj-Napoca, Romania; sonia.socaci@usamvcluj.ro (S.A.S.); anca.farcas@usamvcluj.ro (A.F.); 5 Institute for Conservation and Improvement of Valencian Agrodiversity (COMAV), Universitat Politècnica de València, Camí de Vera 14, 46022 Valencia, Spain; maplaav@btc.upv.es (M.P.); jprohens@btc.upv.es (J.P.)

**Keywords:** *Solanum lycopersicum*, hybrids, physico-chemical evaluation, carbohydrates, phenolic and volatile compounds, volatiles, sensory evaluation

## Abstract

Tomato (*Solanum lycopersicum*) is the globally most consumed vegetable. The objective of this research was to analyze physico-chemical, nutritional and sensorial components (taste and flavor) in two new commercial hybrids (AS 300 F1 and AS 400 F1) and their four F7 parental lines. Two widely grown F1 hybrids (Precos F1 and Addalyn F1) were used as controls. The results obtained for carbohydrates (HPLC-RID) indicated that the highest values (27.82 mg/g) were recorded in the paternal line AS 10 of the new hybrid AS 400 F1. The highest values of total organic acids (HPLC-VWD) were recorded in Addalyn F1 (5.06 m/g), while the highest value of phenolic compounds (HPLC-DAD-ESI⁺) were identified in the maternal line AS 09 of the hybrid AS 400 F1 (96.3 µg/g). Intrinsic sensory values were analyzed by male and female tasters of different ages using a hedonic scale. The tasters’ perception revealed obvious taste differences between tomato genotypes. The study allowed determining genetic parameters of interest (heterosis and heterobeltosis) for the new hybrids, as well as a detailed characterization of the chemical composition and organoleptic quality of the parental breeding lines and their hybrids, which is useful in tomato breeding.

## 1. Introduction

Wild tomatoes are native to the Andean region of Colombia, Chile, Peru and Bolivia [1]. However, there are indications that domestication of cultivated tomato (*Solanum lycopersicum* L.) took place in Mexico [2]. Other researchers have mentioned Puebla and Veracruz as specific places due to the greater number of varieties [3]. The domestication is still unclear but linguistic evidence has postulated Peru and Mexico as the major regions of domestication [4]. Tomatoes are known to be used in cooking in Mexico by the Aztecs by 500 BC and were transferred to the rest of the world by the conquistadors after the capture of the Aztecs’ territory [5]. According to allozyme variation, Rick and Fobes [6] proposed that *Solanum lycopersicum* var. *lycopersicum* (SLL) evolved from *S. lycopersicum* var. *cerasiforme* (SLC). Recently, Blanca et al. [7,8] proposed a two-step domestication process from SLC to SLL based on molecular and morphological evidence. The first step involves the pre-domestication of SLC in the Amazonian region of southern Ecuador and northern Peru. Subsequently, SLC would have migrated to Mesoamerica where it would be domesticated to SLL. Razifard et al. [9] proposed that many traits considered typical of cultivated tomatoes arose in South America. However, these domestication traits were lost or diminished once these partially domesticated forms spread to Mesoamerica, where it was finally morphed into the SLL [2,10]. This domestication and diffusion process was accompanied by a selection of alleles related to fruit color, size and shape and also changes in plant architecture [11,12,13,14]. This process also included various genetic bottlenecks that progressively narrowed the genetic diversity of modern tomato, compared to its wild species [8,15]. Taxonomically, tomatoes belong to the Lycopersicon section of the Solanaceae family, *S. pimpinellifolium* being the closest wild relative, with a divergence of only 0.6% base nucleotide pairs [16].

Common nutrients reported to be present in tomatoes are vitamins, minerals, fiber, protein, essential amino acids, monounsaturated fatty acids, carotenoids and phytosterols [17,18,19,20]. These nutrients perform various body functions including constipation prevention, reduction in high blood pressure, stimulation of blood circulation, maintenance of lipid profile and body fluids, detoxification of body toxins and maintaining bone structure as well as strength [21,22,23].

Sugars, mainly glucose and fructose, represent about half of the dry matter or around 65% of the soluble solids (SS) of ripe tomato fruit. Organic acid in tomato fruits consists mainly of citric and malic acid. The acid content varies from 0.3% to 0.6%. Tomatoes with high acid and low sugar contents are very acidic, while those with high sugar content and low acid content have a bland sweet taste [24]. Reducing sugars (glucose and fructose) are major components of SS; sucrose is also present, but in very small quantities [25]. In general, aroma is a combination of retronasal aroma perception and somatic fiber perception in the trigeminal and taste nerves, which means a combination of volatile and non-volatile compounds contribute to tomato aroma. Flavoring compounds of tomato include sugars (mainly glucose, fructose, sucrose); acids (mainly citric acid and malic acid) and about 400 volatile compounds [26].

Tomatoes are also an excellent source of nutrients and bioactive compounds, com-monly known as secondary metabolites, the concentrations of which are correlated with the prevention of human chronic degenerative diseases, such as cardiovascular disease (CVD), cancer, and neurodegenerative diseases [27,28,29]. Apart from lycopene and other carotenoids, phenolic compounds present in tomato are considered as the primary antioxidants based on their ability to donate hydrogen atoms to reactive free radicals [30]. Phenolic compounds are a large group of plant secondary metabolites which have gained increasing interest because of the growing body of evidence indicating the positive effects of plant derived phenolics on the prevention or the initiation of a large variety of diseases [31,32]. Among the phenolic compounds present in tomato, quercetin, kaempferol, naringenin, caffeic acid and lutein are the most common. Many of these compounds have antioxidant activities and are effective in protecting the human body against various oxidative stress-related diseases [33]. Regarding the protocols most commonly used for the extraction of volatile compounds from tomatoes or other vegetable matrices are those based on headspace sampling [34,35,36,37,38] or solvent extraction [39].

Consumer preferences for tomato fruit and especially for fresh vegetable segments are varied. Most of the time the demand for a product is influenced by the price of the product, the consumer’s income, the socio-economic and demographic factors, and the tastes and preferences of each one. High-quality tomatoes (superior in taste, flavor, juiciness, consistency) attract a loyal consumer who is often willing to pay a higher price for such products (for example, cherry tomatoes, which are high in sugar and tasty). In tomato breeding, an aim is to obtain new cultivars that will satisfy the ever-increasing requirements of the producers and consumers, respectively of the consumer market for fresh fruits and the processing industries. The diversification of the assortment in the direction of quality seems an increasingly important modern desideratum, which cannot be approached with chances of success without a permanent connection to the quality requirements of the consumer, and to the trend that can change in time in this respect.

In the present study, physico-chemical and nutritional-sensory properties of tomato fruits were investigated in two newly F1 commercial hybrids and their four parental lines, and two F1 commercial hybrids (used as controls in the breeding process: Addalyn F1 as a control for new commercial hybrid AS 400 F1, and Precos F1 for AS 300 F1.), in order to examine the relationship between their physico-chemical composition and the data resulting from organoleptic analysis. The intrinsic organoleptic components (taste and flavor) were analyzed by age and sex (men-women) groups of tasters. The study also considered the evaluation of the relation between chemical compounds in fruits in the context of hybrid tomato breeding process. The analysis was performed both on the parental lines and the hybrids resulting from them. Our hypotheses are that different genotypes (parental lines, hybrids) can display be differences in the content of tomato fruits in different useful chemical constituents, as well as differences depending on tasters’ perception of taste and flavor, as important elements of tomato quality. We also intended to test whether these differences can occur only between unrelated genotypes (new commercial hybrids and established hybrids used as control) or also between close related genotypes (new commercial hybrids and their parental lines, obtained by pedigree selection).

## 2. Results

### 2.1. Determination of Carbohydrates and Organic Acids

Significant differences were observed for the glucose content of the fruits on the set of eight genotypes (Table 1). The amplitude of the variation ranged between 5.75 mg/g (AS 300 F1) and 12.52 mg/g (AS 10 ♂ (F7)). The lower limit was registered for one of the two newly created commercial hybrids (AS 300 F1), which presented lower values than both control varieties (Precos F1 and Addalyn F1) and its own parental lines. In contrast, in the other newly created commercial hybrid (AS 400 F1), the glucose content was almost double (11.37 mg/g) the value of AS 300 F1. Based on the glucose content, AS 400 F1 and its parental lines as well as the control Addalyn F1, form a homogeneous group (Table 1). There were no significant differences between their averages, but they all had significantly higher values compared to other genotypes. A similar situation was obtained for the fruit content in fructose, and also for the major types of carbohydrates (glucose + fructose). In both cases, as for the glucose content, AS 400 F1, its parental lines and the control Addalyn F1 constituted a homogeneous group, with higher average values compared to the rest. The amount of glucose plus fructose was between 13.38 mg/g and 27.82 mg/g. The highest values were recorded in the paternal line of the hybrid AS 400 F1, with a value of 27.82 mg/g, followed by its maternal line with the value 27.09 mg/g. The lowest value was recorded in the commercial hybrid AS 300 F1 (13.38 mg/g).

For the organic acids, significant differences were recorded among the eight genotypes for the fruit content in malic acid, citric acid, as well as organic acids: malic plus citric acids (Table 1). The malic acid content ranged between 0.61 mg/g (Precos F1) and 1.73 mg/g (Addalyn F1). Unlike the new commercial hybrid AS 300 F1, the other newly created hybrid, AS 400 F1 gave fruit with much higher content in malic acid. The highest citric acid content was identified in the parent line (AS 10 ♂ (F7)) of the hybrid AS 400 F1 (3.39 mg/g), and the lowest in the hybrid AS 300 F1 (1.73 mg/g). The content in organic acids (as malic plus citric acids) showed a wide variation, ranging between 2.83 mg/g (AS 300 F1) and 5.06 mg/g (Addalyn F1). A relatively large difference was also observed between the two new commercial hybrids AS 300 F1 and AS 400 F1 (respectively 2.83 and 4.33 mg/g).

### 2.2. Determination of Phenolic Compounds

Significant differences were observed among the tomato genotypes for all phenolic compounds (Caffeic-glucoside I, Caffeic-glucoside II, 3-Caffeoyl quinic acid, 5-Caffeoyl quinic acid, Caffeic acid, Ferulic-glucoside, Q-tri glucoside, Q-rutinoside, Naringenin, Ferulic acid) (Table 2). Consequently, total phenolic components (as the sum of all individual phenolic compounds) fluctuated strongly within the eight genotypes. The variation ranged between 38.4 and 96.3 µg/g (for the parental lines AS 30 ♀ (F7) and AS 09 ♀ (F7)). One of the new commercial hybrids (AS 300 F1) had a total phenolic compounds close to those used as control (Precos F1 and Addalyn F1), but lower compared to the other new commercial hybrid (AS 400 F1).

### 2.3. Fingerprinting of Volatile Components by ITEX/GC–MS Analysis

Volatile compounds (73 components) contribute significantly to the differentiation of tomato genotypes ((Supplementary Table S1), for example: hexanal, heptanal, 2-heptenal, 6,10-dimethyl-5,9-Undecadien-2-one, acetophenone, 3-pentanone, 6-methyl-5-hepten-2-one, 3-methyl-1-butanol and 2-methyl-1-butanol display large differences in the set of eight genotypes. In this way, the concentration of hexanal for tomato genotypes ranged from 0.83% to 30.02%. The highest concentrations of hexanal were recorded in the paternal line of the commercial hybrid AS 300 F1, and in the control Precos F1 (30.02% and 29.85%, respectively). The concentration of 3-pentanone for tomato genotypes ranged from 4.37% to 8.20%. The highest concentrations of 3-pentanone were recorded in the paternal line of the commercial hybrid AS 400 F1, and in the commercial hybrid AS 400 F1 (7.32% and 8.20%, respectively).

### 2.4. Correlations between the Pairs of the Main Chemical Compounds

In order to evaluate the connections between the main 17 chemical compounds determined in the tomato fruits, the phenotypic correlation coefficients between all the trait pairs were calculated (Table 3). As expected, very significant positive correlations (*p* < 0.001) were observed between carbohydrates (as glucose + fructose) and fruit content in its components, glucose, and fructose (as well as between glucose-fructose). Additionally, very high positive correlations were recorded between the acid content (as malic + citric acids) and malic acid, and citric acid. A significant and very strong correlation was also recorded between the total phenolic compounds and naringenin. Organic acids (as malic + citric acids) was closely correlated with glucose and carbohydrates, as glucose + fructose (*p* < 0.01). Significant correlations (*p* < 0.05) were registered between the two components of organic acids (as malic plus citric acid) and carbohydrates, as its two components (glucose and fructose). In addition, malic acid was correlated with citric acid, and organic acids (malic + citric acids) with fructose content. Significant correlations (*p* < 0.05) were also recorded between Caffeic-glucoside I and fructose, and carbohydrates, as glucose + fructose; between 5-Caffeoyl quinic acid and Caffeic-glucoside I and Caffeic-glucoside II; and finally, between Q-tri glucoside and 5-Caffeoyl quinic acid and caffeic acid contents.

### 2.5. Organoleptic Tastings—Taste and Flavor Results

**Figure 1:**
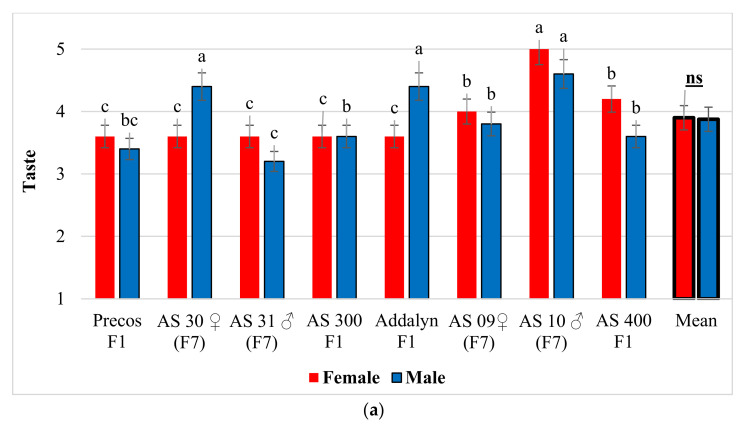

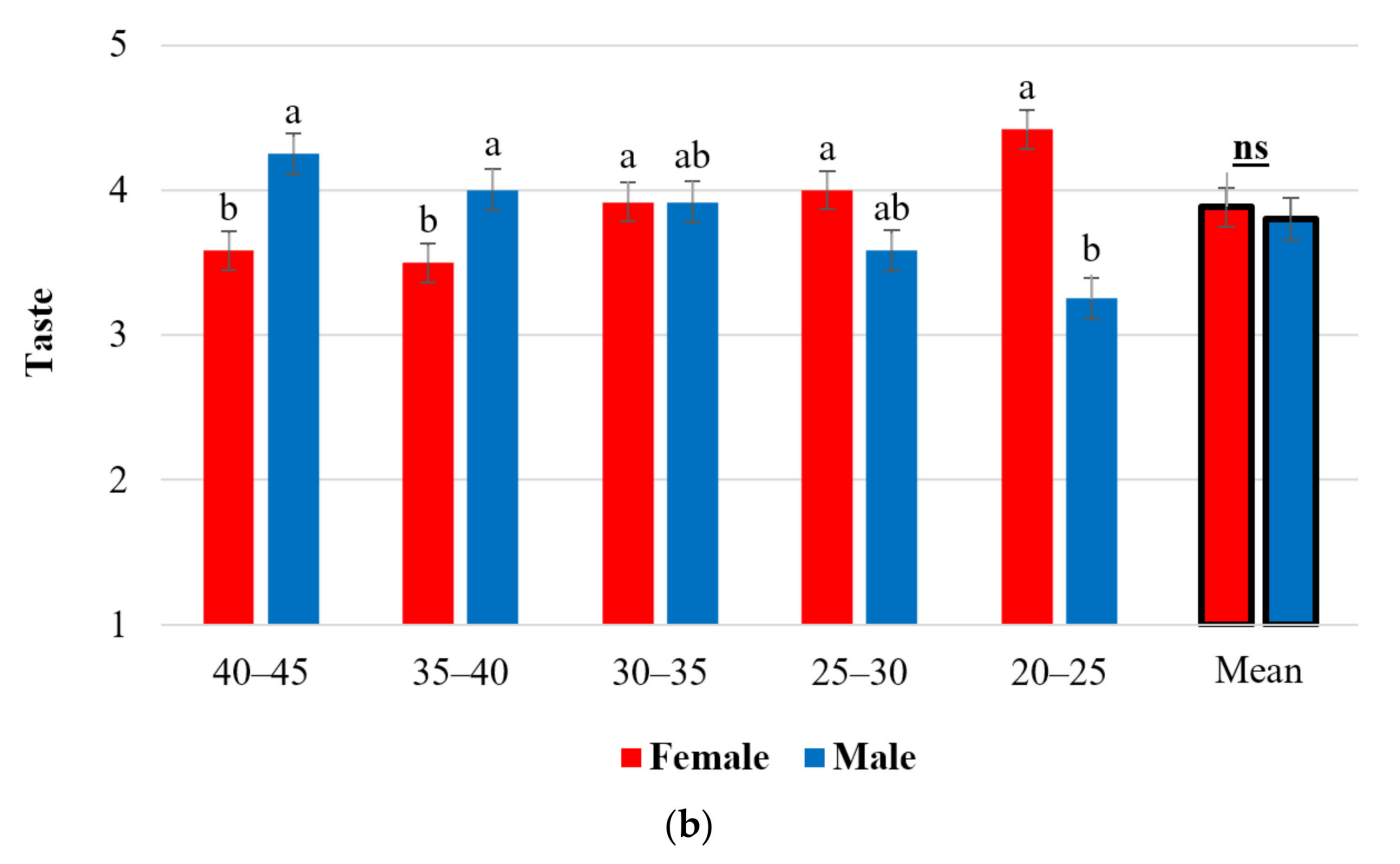
Results obtained for tomatoes taste on a notes scale of 1–5 (1 = weak; 5 = very good): (**a**) Female and male score, depending on genotype; (**b**) Female and male score, depending on the age groups. The notes are expressed as mean ± SD. Any means in the columns followed by the same letter are not significantly different (Duncan test, α < 0.05). On the latest columns, the average values by types of tasters (women-men) were compared using *t*-test (Student), α < 0.05.

Following organoleptic tastings, it was found that significant differences for fruit taste were found among the eight genotypes, both for female and male tasters (Figure 1a). In both categories of tasters, the highest marks for fruit taste were recorded at line AS 10 ♂ (F7). The line AS 31 ♂ (F7) was identified as less tasty, with the lowest marks being given by male tasters. Overall, there were no significant differences in fruit taste of the eight tomato genotypes among male and female tasters.

The perception of tasters by age categories was different for fruit taste (Figure 1b). Real differences were registered in women between the groups of 40–45 years and 35–40 years compared to the groups of 25–30 years and 20–25 years, the last two groups giving significantly higher marks for taste. For men, the 40–45 and 35–40 age groups gave significantly higher marks for fruit taste, compared to the 25–30 age group. When data of women and men were averaged, no significant differences were found among age ranges.

**Figure 2:**
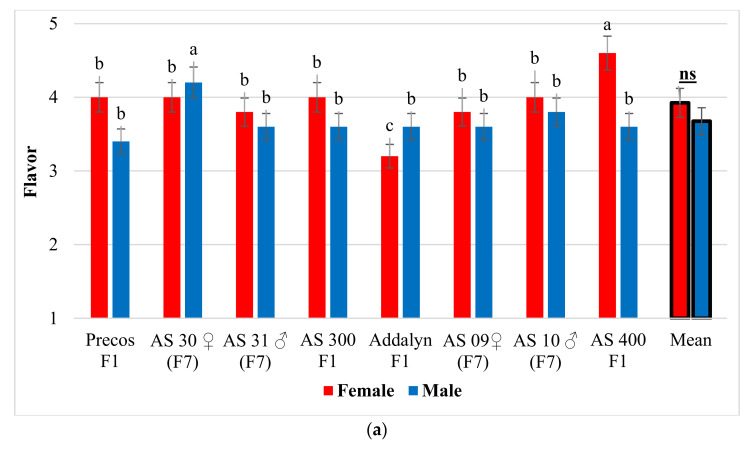

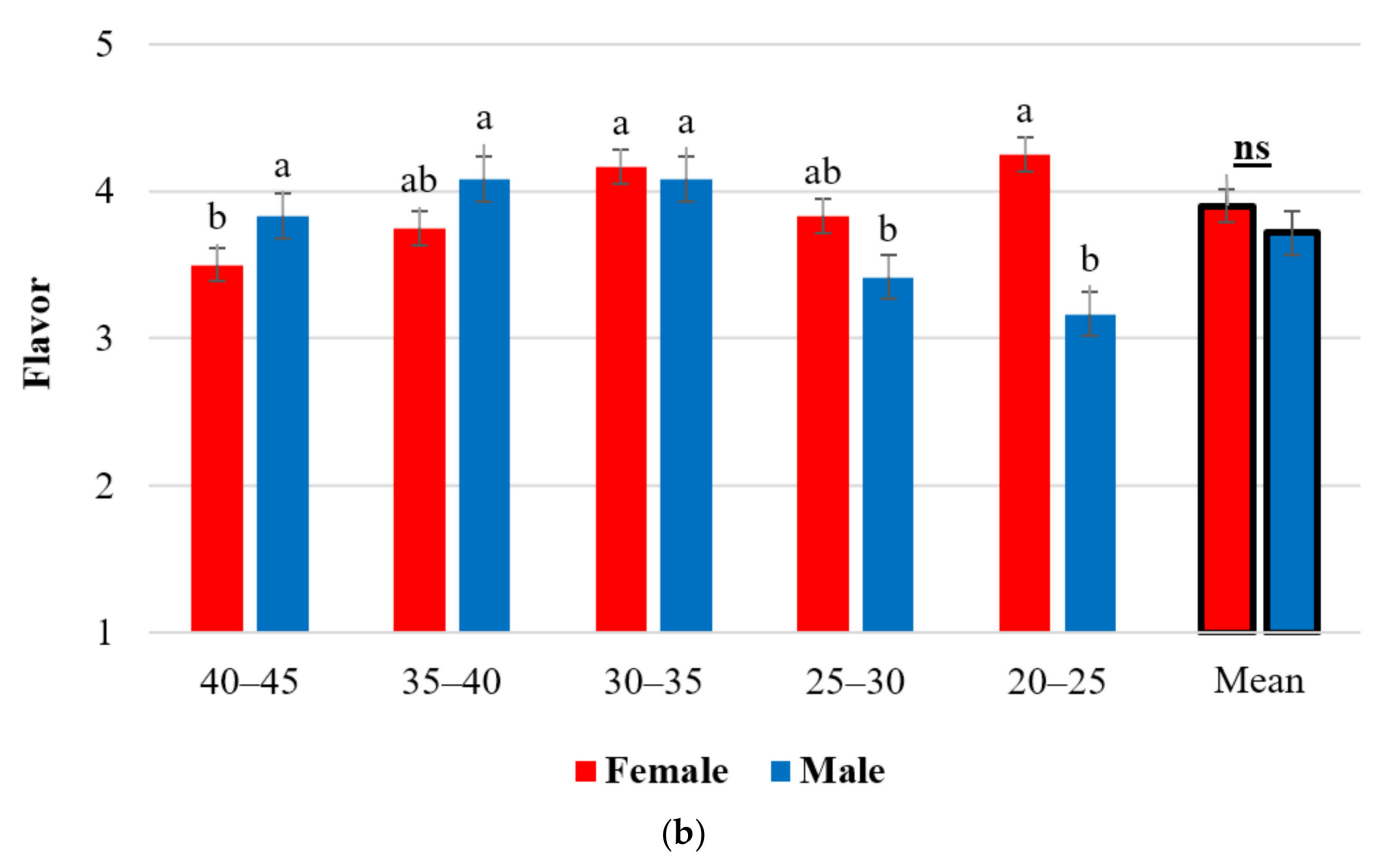
Results obtained for tomatoes flavor on a notes scale of 1–5 (1 = weak; 5 = very good): (**a**) Female and male score, depending on genotype; (**b**) Female and male score, depending on the age groups. The notes are expressed as mean ± SD. Any means in the columns followed by the same letter are not significantly different (Duncan test, α < 0.05). On the latest columns, the average values by types of tasters (women-men) were compared using *t*-test (Student), α < 0.05.

The statistical processing of the marks from the fruit flavor highlighted the different appreciation of the tomato genotypes by the women, while the men did not notice any significant differences between the genotypes analyzed (Figure 2a). Thus, women appreciated the fruit flavor the most at the commercial hybrid AS 400 F1, with a significantly higher value compared to both commercial hybrids used as a control (Precos F1 and Addalyn F1; of these two, Precos F1 obtained significantly higher marks for fruit flavor). In contrast, for the average marks over the entire experience, there were no significant differences in the perception of fruit flavor between men and women.

Regarding the age categories, the appreciation of the flavor was different, both for women and men. Female tasters aged 20–25 and 30–35 gave significantly higher marks than those in the 40–45 age category (Figure 2b). In the case of men, the tasters in the 40–45 and 35–40 years categories appreciated with significantly higher marks the flavor of the tomato fruits compared to those in the 25–30 years and 20–25 years categories. When women men’s data were combined, no significant differences in fruit flavor between women and men, depending on the age of the tasters, were observed.

### 2.6. The Relationships between the Chemical and Organoleptic Parameters

**Figure 3:**
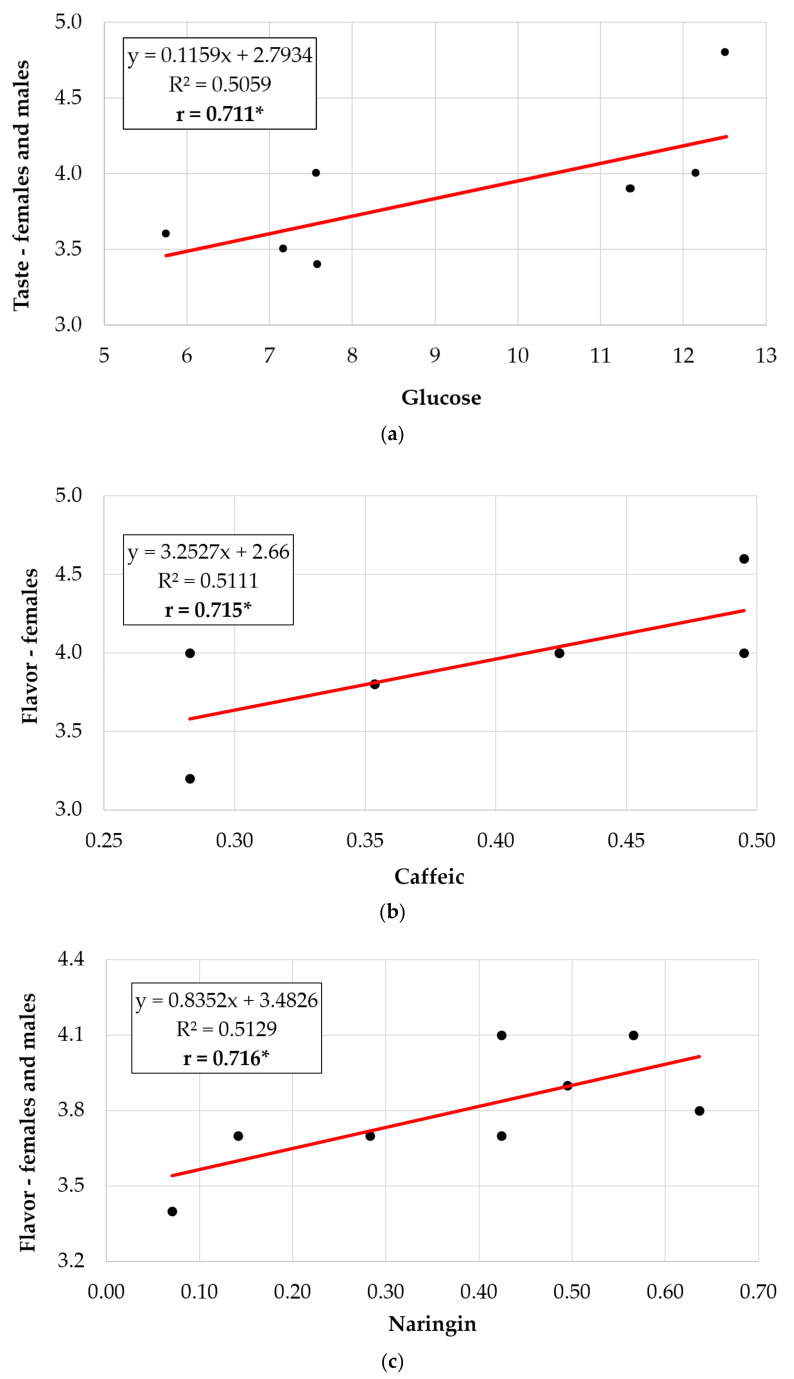
(**a**–**c**) Relations between the chemical contents of the tomatoes and notes on tasting, separately by female and male tasters, and all tasters, as a whole, both male and female tasters, analyzed by regression equations, coefficients of determination and correlation (only the figures for the Pearson correlations with ‘r’ value significant for the 0.05 level are presented).

The relations between the chemical contents of the tomatoes and the marks on tasting, separately by female and male tasters, or with all tasters combined, were also analyzed by computing the regression equations, coefficient of determination and correlation coefficient. Figure 3 contains eight data pairs, although due to overlap of the values of some pairs, Figure 3a appears only with seven points and Figure 3b with six. Tight relations as phenotypic correlations, which were statistically significant (*p* < 0.05), were recorded only between few traits, presented in Figure 3, as follows: the fruit content in glucose and notes for taste given by both male and female tasters and by the grouping of both sexes (Figure 3a); caffeic content and fruit flavor appreciated by women (Figure 3b); and naringenin content and marks for flavor given by all tasters (Figure 3c). In all these cases the calculated ‘r’ was significant, and the line of the regression equation had an ascending, positive value. In addition, the proportion of the variables represented by marks (considered the dependent variable on the chemical content, as an independent variable in regression) from the total variance, was close in all three cases (i.e., 50.59%, 51.11% and 51.29%).

### 2.7. Heterosis

**Figure 4:**
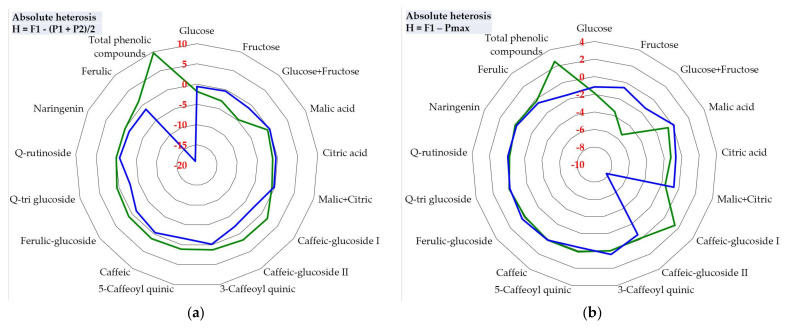

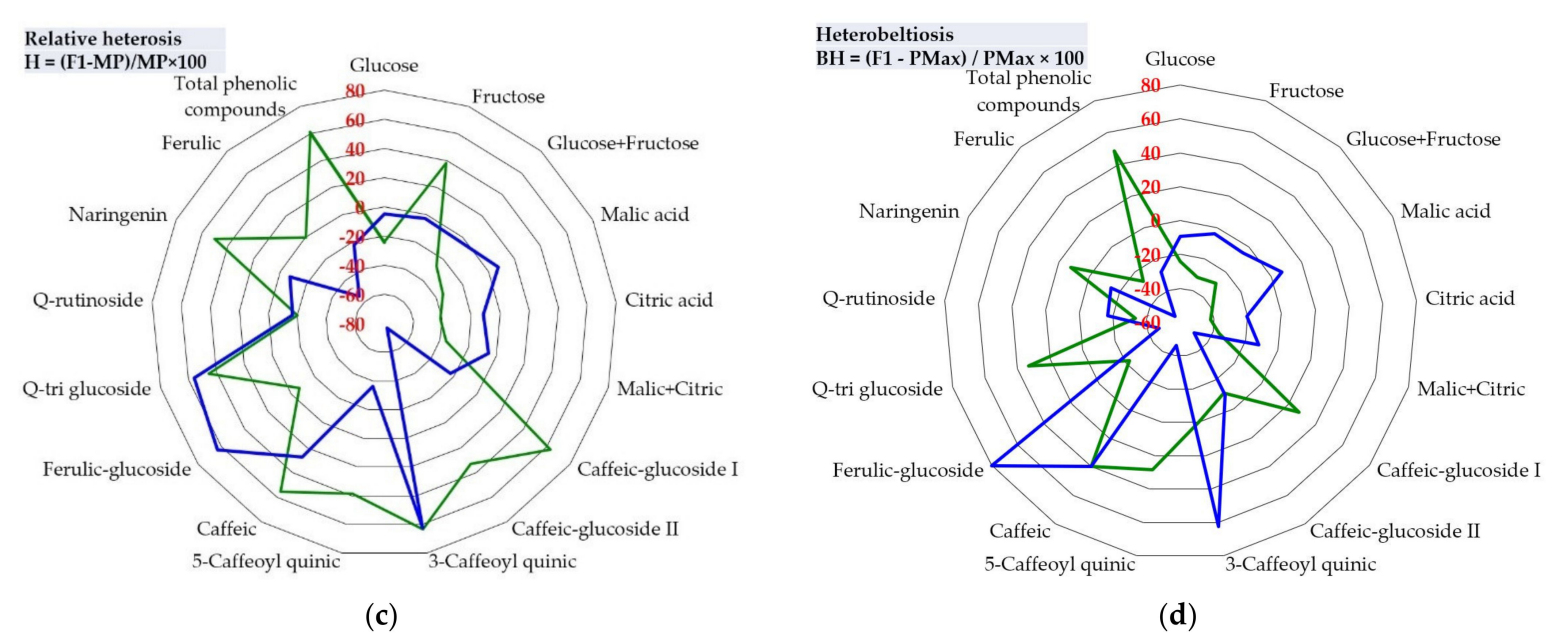
Value of heterosis and its direction (positive or negative) for the main chemical components of tomatoes, depending on the type of heterosis calculated for two newly commercial hybrids and their parental lines in F7: (**a**) Absolute heterosis, based on the average value of the parents; (**b**) Absolute heterosis, based on the average value of the best parent; (**c**) Relative heterosis; (**d**) Heterobeltiosis.

Regardless of the type of calculated heterosis (absolute—using two formulas, relative heterosis and heterobeltiosis), the common feature was the high share of negative heterosis in the new commercial hybrids. Out of all the heterosis values calculated based on the four parental lines and of the 17 chemical elements analyzed, in three types of heterosis the negative values predominated. The heterosis manifested on the two commercial hybrids, AS 300 F1 and AS 400 F1, calculated as the average value of the analyzed traits compared to the average value of the parental lines, displayed high oscillations (Figure 4). Three traits (glucose, citric acid, and malic + citric acids) showed only negative values of heterosis in all combinations of parental lines. No chemical element had only positive heterosis in all four cases.

Different contrasting situations were recorded, sometimes regarding the manifestation of heterosis in the same commercial hybrid (for example, one type of heterosis was positive, but another was negative). Additionally, absolute heterosis calculated by reference to the average of the parents in the two commercial hybrids of ferulic content was positive for AS 300 F1, but negative for AS 400 F1. For total phenolic compounds, a positive absolute heterosis was registered for the commercial hybrid AS 300 F1, but instead, in another commercial hybrid (AS 400 F1) the situation was contrasting, the absolute heterosis being negative (Figure 4a).

The absolute heterosis calculated by reference to the best parent (Figure 4b) does not differ much from the heterosis resulting from the average of the two parents. As in the case of heterosis calculated based on the parental average, most of the values of heterosis were negative. The same three chemical constituents of the fruit (glucose, citric acid, and malic + citric acids) showed only negative heterosis values in the analyzed combinations, only in addition, when ferulic content was added to them.

The negative heterosis of the chemical constituents in fruit, especially for glucose, carbohydrates (as glucose + fructose), malic acid, citric acid, organic acids (as malic + citric acids), Q-rutinoside and ferulic compound) at the two commercial hybrids stands out strongly in the relative heterosis (Figure 4c) and heterobeltiosis (Figure 4d). The results also highlight the differences and different oscillations of heterosis between the two hybrids for the chemical elements in the fruit. Overall, the results (Figure 4a–d) reveal a high share of negative heterosis on most of the 17 chemical components in the fruit.

**Figure 5:**
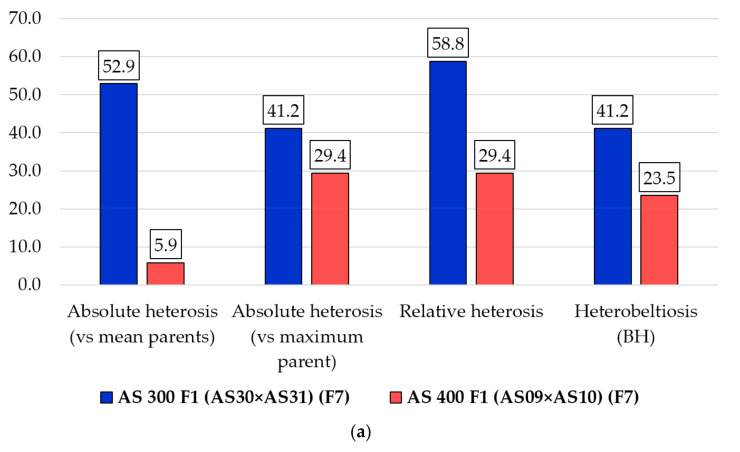

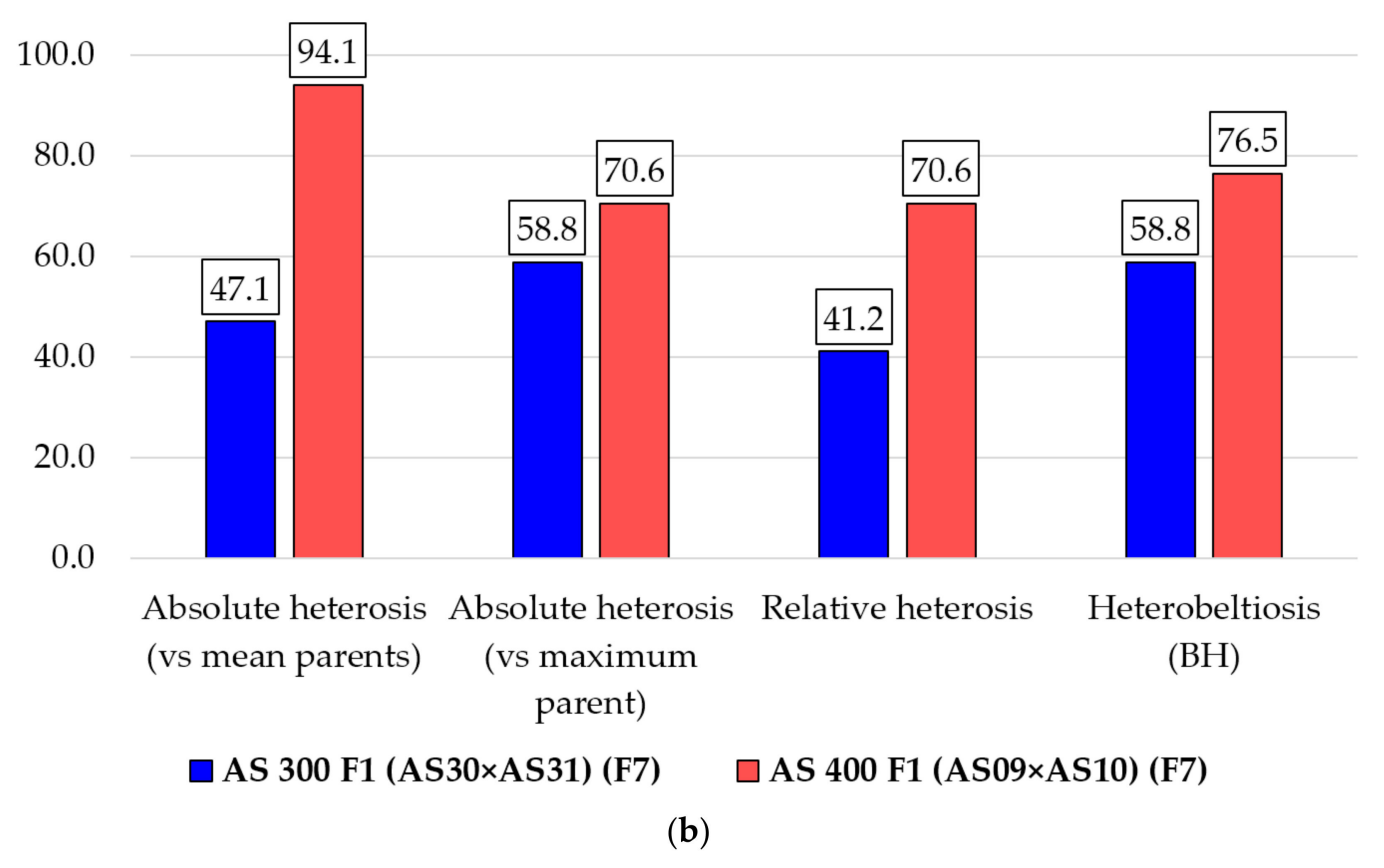
Types of heterosis of the traits represented by the chemical content of tomatoes according to genetic material (parental lines of the two newly created commercial hybrids, AS 300 F1 and AS 400 F1) and the direction of heterosis: (**a**) Positive; (**b**) Negative.

The differences between the values of heterosis were strongly influenced by the genetic material analyzed and the type of heterosis estimated, as it results from the analysis of the entire experience (Figure 5). Thus, the manifestation of heterosis for the chemical content of fruits in the elements analyzed was higher in the commercial hybrid AS 300 F1 than in the AS 400 F1 hybrid.

In addition, in the AS 300 F1 hybrid, two types of heterosis (absolute heterosis calculated by parental mean, and relative heterosis) had a value of over 50%, and the other two types of heterosis had values of over 40%. In contrast, in the AS 400 F1 hybrid, the positive heterosis had much lower values, and fluctuated widely, depending on the type of heterosis calculated, between 5.9–29.4% (Figure 5a). Depending on the type of heterosis calculated, the negative heterosis ranged between 41.2–58.8% at AS 300 F1 and between 70.6–94.1% at AS 400 F1 (Figure 5b). It turned out that on the whole experience, the share of negative heterosis was clearly higher compared to positive heterosis.

### 2.8. Relationships between the Analyzed Parameters

**Figure 6:**
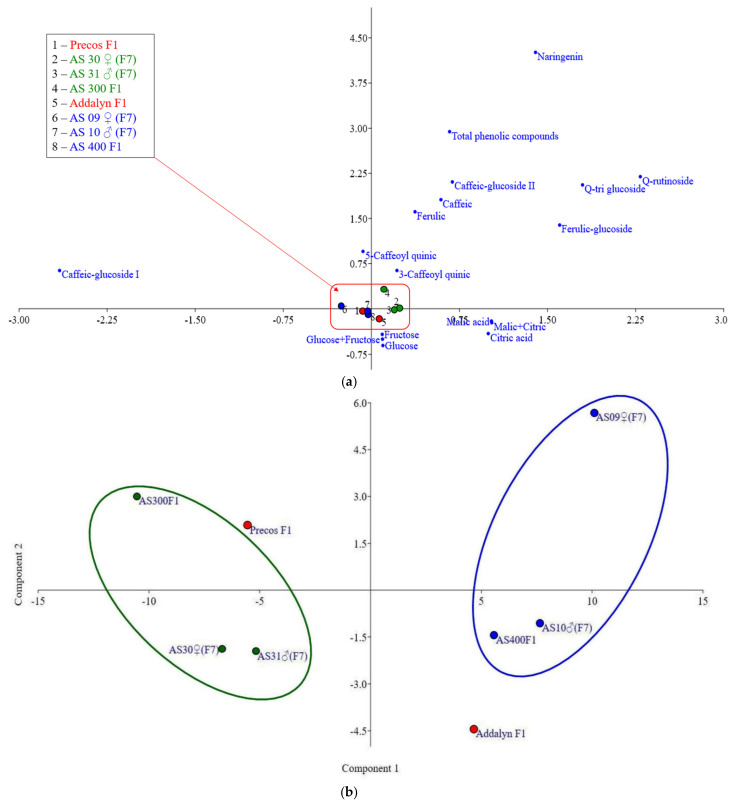

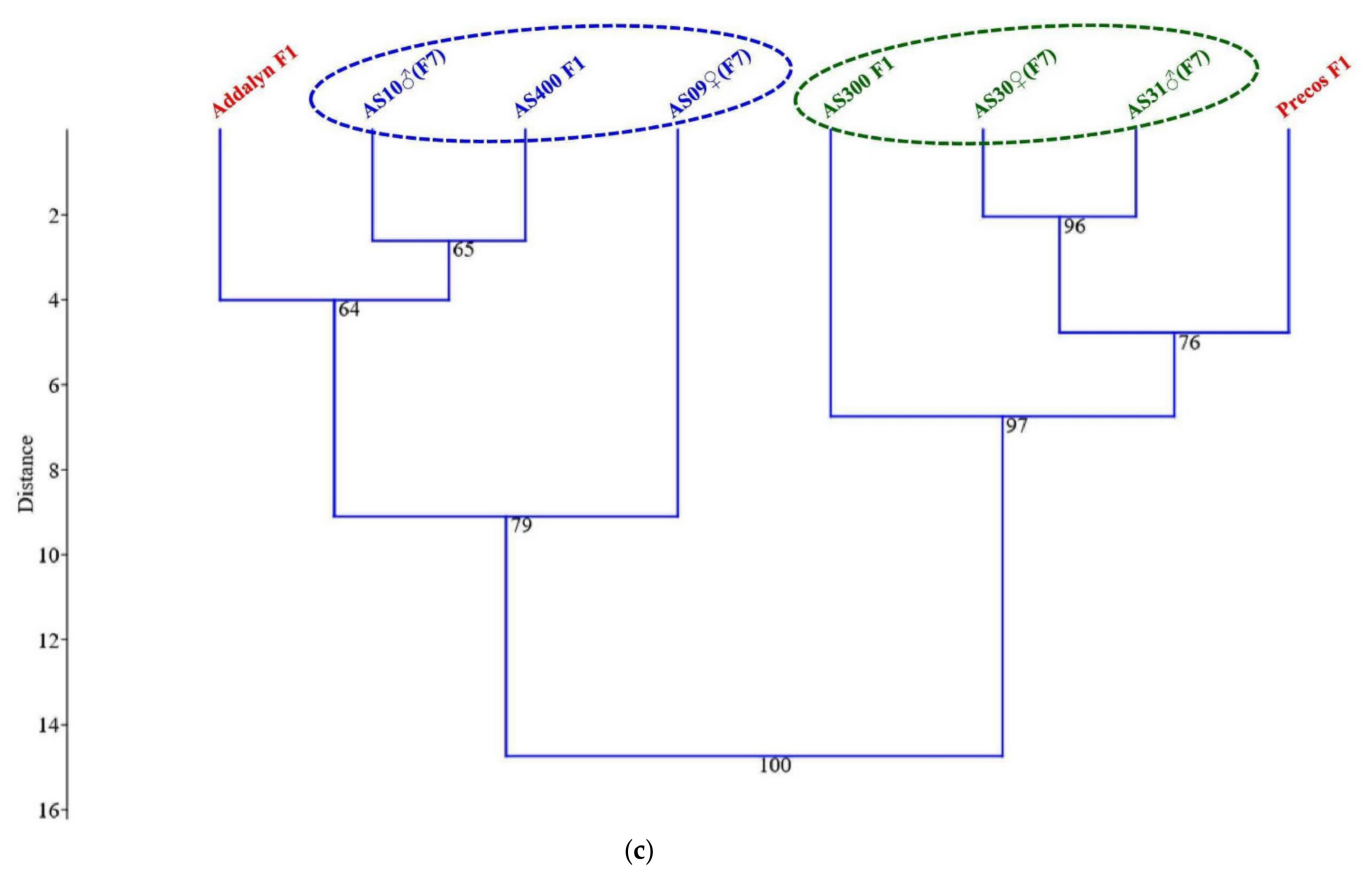
(**a**) Distribution of chemical components in tomato fruits based on correspondence analysis (CA); (**b**) Principal component analysis (PCA) for the two new commercial hybrids (AS 300 F1 and AS 400 F1) and their parental lines; (**c**) Hierarchical clustering—paired group UPGMA (Unweighted Pair Group Method with Arithmetic Mean)—similarity index (Euclidean) of the two new commercial hybrids and their four parental lines (F7), and the two commercial hybrids used as control.

Correspondence analysis (CA) for the chemical content of tomato fruits (Figure 6a) and PCA (principal component analysis) for the two new commercial hybrids and their parental lines (Figure 6b) illustrate their distribution and the extent to which they are correlated with the two main components of the PCA (Figure 6). Overall, the first component of the PCA represented 38.2% of the PCA, and the second component 16.9% of the total variation observed in the matrix analysis of correlation. The data, including hierarchical clustering—paired group UPGMA, similarity index (Euclidean) presented in Figure 6c, confirm that the two hybrids used as control were chosen appropriately: Addalyn F1 as a suitable control for AS 400 F1 new creation, and Precos F1 for AS 300 F1 new commercial hybrid.

## 3. Discussion

### 3.1. Chemical Compounds Variations on Tomato Fruits

Carbohydrate content of the tomato fruit can be influenced by many factors, including environmental conditions and crop technology, method of assessing content, harvest period and genotypes [40,41,42,43,44]. The values for carbohydrates, based on glucose plus fructose levels in this experiment, demonstrate the strong influence of genotype and individual genetic heritage of cultivars on the potential to accumulate a certain amount of carbohydrates. Apart from crop and growth conditions and genotypes, the levels of glucose and fructose, other useful compounds in tomatoes can vary greatly depending on some stressors [40,45]. The stress factors can be represented by water, saline, thermal stress, etc. In order to be able to obtain high values in terms of all the carbohydrates in the tomato fruits, except genotypes, special attention must be paid to the environmental factors and to the growth of the fruit in optimal cultivation conditions [46,47].

The values recorded for the analysis of organic acids were high compared to other experiments. In some works, the reported values were very low, i.e., between 0.36–0.55 mg/g [48] about ten times lower than other references. In addition, we also observed that the concentration of sugars and organic acids was correlated with some important volatile compounds compared to other research [49].

Typical results for citric acid in tomato fruits are 5.41–8.06 g/kg [50], 5.00–10.00 g/L [51], 3.94–7.11 g/L [52]. Generally, citric acid concentration represented the 40–90% of the total amount of organic acids, as malic acid + citric acid, while the malic acid concentration ranged from 10–60% of citric acid concentration [53].

The total phenolic content calculated as the sum of the individual phenolic compounds in each genotype had values between 38.4 and 96.3 µg/g. The highest values were recorded by the group of 3-Caffeoyl quinic, 5-Caffeoyl quinic, which are part of chlorogenic acids that are phenolic acids with neighboring hydroxyl groups. These are aromatic residues derived from the esterification of cinnamic acids, including caffeic, ferulic and *p*-coumaric acids with quinic acids. Caffeic acid is one of the most abundant phenolic acid in tomato fruits [54,55]. The most abundant flavonoid in the tomato genotypes studied in other research [56] was quercetin pentosylrutinoside; the highest value was recorded in yellow tomato with a high level of phenolic compounds (54.23 µg/g), including phenolic acids (43.30 µg/g) and flavonoids (10.93 µg/g). Naringenin, which is also a flavonoid, indicated lower values than other results [57]. Phenolics in tomato fruits, represented by chlorogenic acid and quercetin [58] are important antioxidants both for plant integrity and human health [59].

For volatile compounds extracted from tomato juice, the highest values were compared to the results obtained by Wang et al. [60] who concluded that the highest concentrations of volatile compounds are in the pericarp compared to the locular gel. These volatile compounds were biosynthesized from various pathways in the tomato. Hexanal recorded the highest value in our study- Other results reported [61] prove that hexanal was generated from C18 fatty acids, which conferred “grassy”, “tallow”, “fatty” notes to the tomato. Additionally, 3-methyl butanol and 2-methyl butanol were biosynthesized via the removal of amino groups from amino acids by branched chain aminotransferases (BCATs) [62].

Aldehydes (hexanal, cis-3-hexenal, trans-2-hexenal) are released from vegetative tissues and they provide a fresh, green character to the tomato aroma; ketones (acetone, geranylacetone and β-ionone), on the other hand, contribute to the fruity aroma [63]. The compounds 6-methyl-5-hepten-2-one (found in parental lines of AS 300 F1 (F7) and Addalyn F1 control), geranylacetone, β-ionone are derived from carotenoids by enzymatic cleavage [64]. The compound 6-methy1-5- hepten-(Z)-one is responsible for the sweet or floral note in the tomato aroma, while geranylacetone is related with the sweet, citrus or ester aroma in tomatoes; both compounds are known as lycopene degradation products [65]. Other reported analysis found that the effects of aldehydes were positive, while cis-3-hexenol, hexanal and apocarotenoids had a negative effect on fresh tomatoes [66].

The result indicates that naringenin is one of the most important flavonoids present in tomato fruits generally, but also in this experiment, not only in the skin [67] and its role and therapeutic potential is extremely well known and recognized [21,68,69].

### 3.2. Sensory Analyses

The study for sensory analysis was performed to define and compare the differences in taste and flavor between the genotypes analyzed. From the results of the panel tests, sensory profiles were designed for each genotype. A clear difference was observed between the profiles of the two control varieties: Precos F1 control registered the average values at the sensory parameters (taste, flavor). Some specific characteristics were highlighted, especially for the breeding hybrids: for taste the improved hybrids AS 300 F1 and AS 400 F1 indicated lower values compared to the parents.

Acceptable fruits must be rich in sweetness and with a typical aroma of the tomatoes, but intermediate in acidity [70]. A crucial aspect will be the definition and understanding of the chemical compounds responsible for the perceived specific flavors. The synergistic effects between glucose and citric acid are of interest, because the concentration of citric acid seems to influence the perception of sweetness, increasing the importance of glucose compared to fructose [71].

Sensory analysis clearly evidenced differences among genotypes and differences between new hybrids and their parental lines. Compared to the results obtained by Mikkelsen (2005) [72], where the average values reported for flavor were between 2.7–3.4, the newly hybrids registered values higher than 3, which can be considered valuable in terms of intrinsic organoleptic characteristics.

Many factors can affect the final sensory analysis: climatic and cultural conditions can affect tomato flavor for example, heavy rains prior to harvest appear to dilute the concentrations of flavor compounds [73], harvesting and handling techniques impact the flavor of the ripened tomato fruit [74,75], and temperatures below 16 °C may influence tomato flavor by lowering the volatile content and reducing “tomato-like” flavor [76].

In some studies, at fruit tasting sessions, there were different perceptions for the taste and flavor of fruits between tasters (for example, at apples), depending on the gender [77]. It has been hypothesized that women probably have a finer perception of taste and flavor compared to men.

However, among tasters there is a consistency in terms of taste appreciation depending on the soluble substances: carbohydrates and their main components (glucose and fructose) directly and positively influence the taste of tomatoes, for both female and male tasters. But the regression line, the coefficients of determination and correlation indicate that women prefer sweeter fruits compared to men. Probably the situation is similar to that found in apples, when females disliked the sour-astringent fruits, while males liked acidulated fruits [77]. The conclusion may be also that females give higher importance ratings of fruit flavors than males [78].

The sensory analysis clearly highlighted the differences between the genotypes and the differences between the new commercial hybrids and their parental lines. The total values of the levels of chemical compounds demonstrate the strong influence of the genotype and individual genetic inheritance of the hybrids on the potential of tomatoes to accumulate a certain amount of carbohydrates, organic acids, phenolic compounds and volatile compounds. Perception of some taste descriptors, such as taste as a whole, sour or sweet, can be influenced by the natural levels of volatile substances probably more depending on the individual than by a group of individuals grouped by different sexes or different ages.

The tasters were informed that the results showed that women aged 20–35 indicated higher values for the taste of tomatoes compared to other groups of women, and a similar situation was registered in men, in those aged 40–45 years. In the discussion sessions, the participants considered that each taster had a different individual perception, given by their own sense, received and rendered with the help of the taste buds, as receptor cells, located on the tongue. They agreed that they can fit into a general model, according to which about a quarter of people have a super-tasting ability due to which they feel the taste more intense than the rest.

### 3.3. Genetic Parameters and Their Utility in Tomatoes Breeding

Heterosis is a classic way of analyzing the hybrid vigor obtained from crosses between genetically different parents. Its use remains extremely topical in genetics and plant breeding, including in tomato breeding [79,80]. It is generally accepted that the heterosis effect is manifested when an F1 hybrid is superior to its parents in terms of plant growth, production capacity, response to stressors and adaptability, etc. However, in tomatoes, as in other plants, heterosis can be positive or negative, depending on the direction in which a given trait is manifested in F1, respectively if it is superior to the stronger parent or inferior to the weaker parent. In fact, heterosis can be observed in F1 generation in respect to one or more traits, as well as in respect to single morphological, physiological, or biochemical traits [81]. Heterosis manifests differently in the individual F1 combinations, depending on the value of the cultivars or parental lines, and their general combining ability (GCA) and specific combining ability (SCA) [80,82]. However, heterosis effects cannot be exactly predicted beforehand, but sometimes the probability of predicting it could be very high, even more than 90% [81].

The current study provides evidence that overdominance can play the important role in tomatoes heterosis of useful chemical components in fruits, as stated by other researchers for different quantitative traits [83,84,85]. Despite all the difficulties noted in the present study in obtaining positive and significant heterosis for chemical elements of interest in tomatoes fruits, there are favorable premises for the tomato breeding and improvement of tomatoes’ economic and quality importance. This research highlights the assertion that the genetic and biochemical bases of heterosis are far from clear, but its commercial exploitation is “quite rewarding” [86]. Research in this area is still needed, and being up to date is particularly useful. The best argument in this regard is that tomato hybrids have largely replaced the ’classical’ varieties, in the whole world, precisely because of heterosis effects.

### 3.4. General Considerations and Verification of Hypotheses—Judicious Choice of Cultivars used as Control, the Influence of Genotype on the Chemical Content of Useful Substances in Tomatoes and Conclusions of Organoleptic Tests

Controls were used in the study to compare the own creations (new commercial hybrids, obtained at the Agrosel Research Station, Romania) with commercial hybrids recognized for their superior quality, which dominates the autochthonous market in Romania. The Precos F1 hybrid was chosen because it is one of the most cultivated tomato hybrids, especially in southern Romania, where the largest vegetable producers are located. It was also chosen for two other reasons: for extra-early maturity and for the superior organoleptic quality of the fruits. Precos F1 was considered an adequate control for the AS 300 F1 hybrid, which has valuable traits, including productivity, but also properly response to disease attack, i.e., fusarium wilt, verticillium wilt, and nematodes (peculiarities not found in the hybrid used as a control). For the new commercial hybrid AS 400 F1, it was considered that Addalyn F1 can be a suitable control. Addalyn F1 is known and appreciated by Romanian growers and consumers. The aim was to identify possible differences between the two cultivars for different quantitative and qualitative parameters, including resistance to stress factors, productivity, fruit size and quality, tolerance to the tomato bronze spot virus (tomato spotted wilt virus—TSWV), etc.

The data obtained and especially the PCA analyses showed that the two commercial hybrids used as a control were properly selected, including for the purpose of this research: analysis of fruit content in useful compounds, of interest for the nutritional and gustatory quality of tomatoes.

The genotype clearly contributes to the appearance of significant differences both for the content of tomatoes in different useful substances (carbohydrates, organic acids, phenolic compounds, volatile compounds) and to differences in consumers’ perception of fruit quality (taste and flavor).

However, correspondence analysis, principal component analysis, and the dendrogram constructed based on UPGMA algorithm suggestively illustrates how close the two newly created hybrids are to their parental lines regarding the genetic constitution that determines the chemical content of the tomatoes. This closeness is obvious, even if their parental lines were chosen from a different initial breeding material, with different origins, to later ensure the premises for heterosis. In addition, based on these data and as previously mentioned, it is evident that the two hybrids were properly chosen as a control, not only for many characteristics desired in the practical process of creating new cultivars, but, as it turned out in the present research, also for the chemical composition of the fruits.

Negative heterosis, which predominated for most of the chemical components in the analyzed tomato fruits, denotes the difficulty of the process of tomato breeding for fruits with increasingly rich content of useful chemical compounds, which is of great interest for the nutritional and gustatory value of fruits. Nevertheless, a pertinent choice of biological material used in tomato breeding can ensure successful premises in creating new tomato hybrids with particular qualities and appropriate to market requirements, consumers and tomato processors.

Even if some differences have been identified in the tasting of tomatoes on different groups of tasters, women and men, or different age groups, it seems that the individual option decisively dictates the consumer’s orientation for a certain type of product. Breeding tomatoes must have a prospective character, with the tastes and the tendency of the consumers for a certain type of tomato being able to change in time. There are many factors that can act in this regard, i.e., socio-economic, psychological, educational, cultural, etc.

Organizing degustation tests to prospect consumer preferences can be extremely useful for possible identification of new directions for research and breeding of tomatoes. Producers can consider such studies, orienting themselves for a general direction (tomato cultivars to the liking of most consumers), or cultivars to be intended for a ’target’ group, more numerically limited, but which can be of economic interest for a ’directed’ production, i.e., higher price for a more selective category of consumers.

## 4. Materials and Methods

### 4.1. Cultivation Conditions and Plant Material

**Figure 7:**
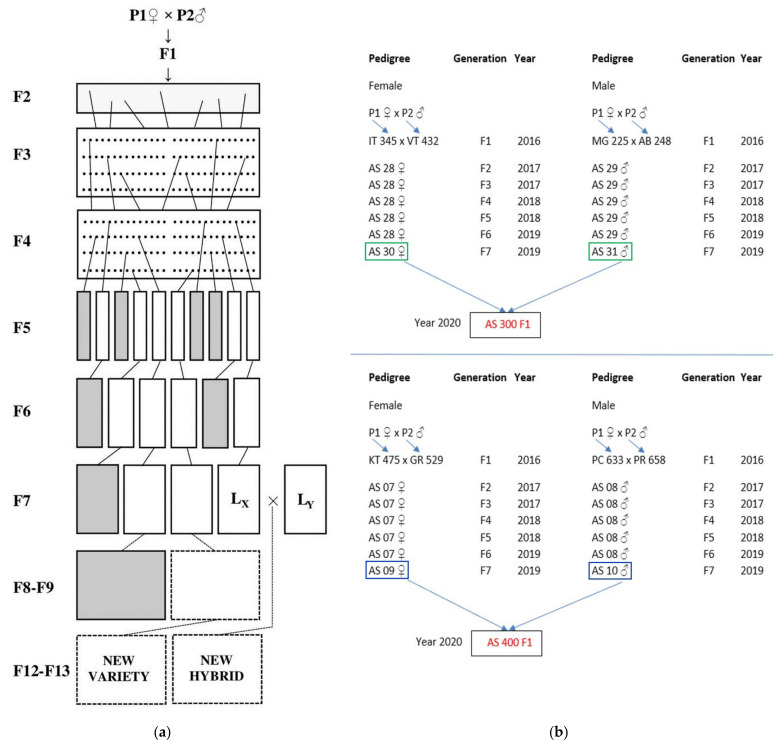
(**a**) ‘Classic’ scheme—pedigree selection, widely used to obtain new tomato varieties. The scheme can also be used for the selection of new lines, which after testing GCA and SCA values can be used as parental forms for obtaining commercial hybrids; (**b**) Scheme for obtaining the two newly commercial hybrids used in experiment: AS 300 F1, and its parental, maternal and paternal lines AS 30 ♀ (F7) and AS 31 ♂ (F7); AS 400 F1 and its parental, maternal and paternal lines, AS 09 ♀ (F7) and AS 10 ♂ (F7). The parental lines were obtained based on the scheme of Figure 7a, up to F7 generation.

The experiment was performed at the Agrosel Research Station, in Câmpia Turzii, N-W of Romania. Tomato genotypes were grown in an unheated solarium (oriented in the south–north direction), in the spring-summer season, in long cycle conditions. The solarium had an area of 240 m^2^, equipped with black agrotextile, an automated fertigation system ITU Mix Station 300 (Itumic Oy, Finland), an automated humidification system K-Rain RPS 1224 (Budapest, Hungary), a reverse osmosis irrigation system for irrigation water HIDROFIT (Mineralholding Kft, Budapest, Hungary) and an Agrosense Base weather station (Sys-Control Kft, Budapest, Hungary). The plant material was represented by eight tomato genotypes, of which two were newly developed commercial hybrids obtained at the Agrosel Research Station (AS 300 F1 and AS 400 F1), and their four parental lines, which are advanced generations of selection from a pedigree breeding process (Figure 7). Two successfully established commercial hybrids (Precos F1—obtained from Geosem, Bulgaria, and Addalyn F1—Hazera Seeds, Netherlands) were used as controls. All genotypes had medium to large fruits (with an average weight between 90–230 g).

All parental lines were coded with ‘AS’ and the maternal or paternal parent symbol (♀, ♂) depending on the parental formula in which they were crossed to obtain commercial hybrids (Figure 7a). The scheme for obtaining the two commercial hybrids (AS 300 F1 and AS 400 F1), created at the Agrosel Research Station, is presented in Figure 7b.

The plant material in the two commercial hybrids was different in terms of the types of tomatoes. For the AS 300 F1 newly hybrid, the maternal line had semi-determined growth, and the paternal line had determined growth (*validum* type). This hybrid was similar in general characteristics to the Precos F1 hybrid used as control, being comparable for different traits of interest, including earliness and yield. Parental lines of AS 400 F1 newly hybrid were of semi-determined growth type. This hybrid had some similarities to Addalyn F1 for multiple traits of interest, including fruit size, resistance to diseases and firmness (LSL-long shelf life). The chemical and organoleptic characteristics of the fruits were performed to compare the eight genotypes.

### 4.2. Chemicals and Equipment

The determination of organic acids was performed by HPLC-VWD (High Performance Liquid Chromatography -Variable Wavelength Detector) method, carbohydrates with HPLC-RID (High Performance Liquid Chromatography-Refractive Index Detector) where data acquisition and interpretation of results was performed using OpenLab software—ChemStation (Agilent Technologies, 95052, Santa Clara, CA, USA). Determination of phenolic compounds was performed by HPLC-DAD-ESI (High-Performance Liquid Chromatography–Diode Array Detection–Electro-Spray Ionization) + method (with acidified methanol, acetic acid, acetonitrile) plus the Agilent 1200 HPLC system equipped with quaternary pump, solvent degasser, autosampler, UV-Vis detector with photodiode (DAD) coupled with mass detector (MS) singlequadrupole Agilent model 6110 (Agilent Technologies, 95052, Santa Clara, CA, USA). Extraction and then separation of volatile compounds were performed using the ITEX/GC-MS technique (“in-tube extraction” coupled with gas chromatography and mass spectrometry), using a GC-MS Shimadzu equipment model QP-2010 (Shimadzu Scientific Instruments, Kyoto, Japan) equipped with Combi-PAL AOC-5000 autosampler (CTC Analytics, Zwingen, Switzerland), as well as CaCl_2_, N_2_, helium.

### 4.3. Composition Traits

#### 4.3.1. Determination of Carbohydrates

Two g of crushed sample were extracted with 4 mL of double-distilled water, followed by vortexing Heidoph Reax top, sonication 30 min Elmasonic E 15 H, centrifugation at 8000 rpm for 10 min and T = 240 °C. The supernatant was filtered through a Chromafil Xtra PA-45/13 nylon filter 0.45 µm and 20 µL extract were injected into the HPLC (High-performance liquid chromatography) system. Agilent 1200 HPLC system equipped with quaternary pump, solvent degasser, manual injector coupled with refractive index detector (RID) (Agilent Technologies, Santa Clara, CA, USA) was used to quantify carbohydrates. The compounds were separated on a Polaris Hi-Plex H column, 300 × 7.7 mm (Agilent Technologies, Sabta Clara, CA, USA), using 5 mM H_2_SO_4_ mobile phase with a flow rate of 0.6 mL/min, column temperature T = 800 °C and RID temperature T = 350 °C. Interpretation of the results was performed using OpenLab-ChemStation software (Agilent Technologies, Santa Clara, CA, USA).

The identification of the compounds in the analyzed samples was done by comparing the retention times with those of the standard compounds. Retention time (Rt) was expressed by standard carbohydrate chromatogram: glucose Rt = 10.22 min and fructose Rt = 10.87 (Supplementary Figure S1). Two calibration curves were made for the quantification of carbohydrates (Supplementary Figure S2).

#### 4.3.2. Determination of Organic Acids

Two g of crushed sample were extracted with 2 mL of double-distilled water, followed by vortexing Heidoph Reax top, sonication 30 min Elmasonic E 15 H, centrifugation with Eppendorf AG 5804 centrifuge. The supernatant was filtered through a nylon filter Chromafil Xtra PA-45/13 0.45 µm and 20 µL extract were injected into the HPLC system. Agilent 1200 HPLC (High-performance liquid chromatography) system equipped with a quaternary pump, solvent degasser, manual injector and UV-VIS detector (VWD) (Agilent Technologies, Santa Clara, CA, USA) was used to quantify organic acids. The separation of the organic acids was done on an Acclaim OA Dionex (Thermo Fisher Scientific, Waltham, Massachusetts, United States) chromatographic column measuring 4 × 150 mm, with 5 µm particles, which was eluted with 50 mM mobile NaH_2_PO_4_ and pH = 2.8 for 10 min at a temperature of 200 °C, with a flow rate of 0.5 mL/min. The results were interpreted with Agilent ChemStation software. Malic and citric acid standards (CheMondis GmbH, Köln, Germany) were used for quantification.

The HPLC chromatogram for organic acids was recorded at the wavelength λ = 210 (Supplementary Figure S3). Retention time (Rt) and absorption spectrum (λmax), were expressed by standard chromatogram: malic acid Rt = 3.08 min; citric acid Rt = 3.32 min (Supplementary Figure S4).

#### 4.3.3. Determination of Phenolic Compounds

Three g of crushed sample were extracted with 2 mL of acidified methanol with 1% concentrated HCl, vortexed for 1 min on Heidoph Reax top, sonicated for 60 min with Elmasonic E 15 H, centrifuged with an Eppendorf AG 5804 centrifuge. The supernatant was filtered using nylon Chromafil Xtra PA-45/13 0.45 µm and 20 µL extract were injected into the HPLC system. An Agilent 1200 HPLC (High-performance liquid chromatography) system equipped with quaternary pump, solvent degasser, autosampler, UV-Vis detector with photodiode (DAD) coupled with Agilent singlequadrupole mass detector (MS) model 6110 (Agilent Technologies, Santa Clara, CA, USA) was used. The separation of the compounds was performed on an Eclipse XDB C18 column, dimensions 4.6 × 150 mm, with 5 µm particles (Agilent Technologies, Santa Clara, CA, USA), using mobile phases A and B in the gradient below, for 30 min, at a temperature of 250 °C, with a flow rate of 0.5 mL/min. Solvent A: Water + 0.1% Acetic Ac, solvent B: Acetonitrile + 0.1% Acetic Ac. Chromatograms were recorded at wavelength λ = 340 nm. For MS, the positive ionization ESI mode was used in the following working conditions: capillary voltage: 3000 V; temperature: 3500 °C; nitrogen flow: 8 L/min; *m/z*: 100–1200, full-scan. Data acquisition and interpretation of results were done using Agilent ChemStation software.

Spectral values were recorded in the range 200–600 nm for all peaks, and chromatograms were recorded at the wavelength λ = 340 nm. For the retention time (Rt) values were recorded between 10.87–17.32 min, and the mass spectrum [M + H] ^+^ had values between 181–743 *m/z* (Supplementary Table S2). Calibration curves for total phenolic content with chlorogenic and rutin standard of 99% purity, with ug/mL concentration ((a) chlorogenic R² = 0.9937; (b) rutin R² = 0.9981, *p* < 0.05) mentioned in (Supplementary Figure S5).

### 4.4. Sensory Characteristics

#### 4.4.1. Taste and Flavor Evaluation

The sensory evaluation of the fruits was performed with voluntary participants, non-experts in the field, by individually completing organoleptic tasting sheets. The tastings were performed on five dates, corresponding to different harvests, and each time the fruits were harvested on the day of evaluation. Each tomato genotype was harvested and evaluated every 10 days. The fruit tasting took place between 15:00 and 17:00, so that the participants were able to eat and drink water two hours before the evaluation. People rinsed their mouths with distilled water before tasting each sample of tomatoes. Each participant received for evaluation ripe tomato fruit. Tasters were invited to assess the quality of the fruit without being provided with information on the samples, instead being provided with brief information on how the notes (marks) are given, on a hedonic evaluation scale, from 1 to 5, for each analyzed trait (1 = low level of the analyzed trait; 5 = high level of the analyzed trait). The same tasters participated in all tasting rounds. The participants were framed into groups of 20 people for each category, respectively five age groups (between 20–50 years) and sex (women and men). The results obtained were subsequently processed as average values of the notes awarded on each trait and group of participants. The evaluation by the tasters included several traits, including fruit size, shape, colour, consistency, juiciness etc., but in this research, we included only the results of analyzing the sensory traits (taste and flavor) of each genotype.

#### 4.4.2. Extraction of Volatile Compounds

Extraction and then separation of volatile compounds were performed using ITEX/GC-MS (‘in-tube extraction’ coupled with gas chromatography and mass spectrometry), using Shimadzu GC-MS equipment model QP-2010 (Shimadzu Scientific Instruments, Kyoto, Japan) equipped with AOC-5000 Combi-PAL autosampler (CTC Analytics, Zwingen, Switzerland) and ZB-5ms capillary column, 30 m × 0.25 mm id × 0.25 µm (film thickness) (Phenomenex, Torrance, CA, USA). Each sample was placed in a 5 g ampoule headspace, together with 2 mL of CaCl_2_ solution. The vial was sealed and incubated at 60 °C for 20 min with continuous stirring. The volatile compounds accumulated in the headspace phase of the ampoule were adsorbed, using a syringe, into a Tenax fiber (ITEX-2TRAPTXTA, (G23)-Siliconert 2000, Tenax ta 80/100 mesh, Switzerland) and then thermally desorbed in gas chromatograph injector. After each analysis, the fiber was cleaned by passing a warm stream of N_2_. The temperature program for the chromatographic column was as follows: maintenance for 10 min at 35 °C, followed by an increase to 50 °C at a rate of 3 °C/min and then an increase to 150 °C by 6 °C/min. Finally, the column was brought to 200 °C and kept at this temperature for 5 min. The carrier gas was helium, at a constant flow rate of 1 mL/min. The injector temperature, ion source and interface were set at 250 °C. The MS detector was used in electronically impact ionization mode in a scanning range of 35–350 *m/z*. The split ratio was 1:5. Separate volatile compounds were identified by comparing the mass spectra obtained with those in the NIST27 and NIST147 mass spectrum libraries [87].

### 4.5. Statistical and Multivariate Data Analysis

For the content in different chemicals, analyses were performed on fruits from two plots (5–6 plants/plot) on each genotype. Within each genotype, two or three fruits were harvested per plant, which were homogenized in the form of a crushed sample of 100 g. From this, a homogenized composition was extracted for chemical analysis, depending on the type of analysis, i.e., two or three grams per analysis. The data were processed as the mean of the traits and standard deviation (SD). Analysis of variance (ANOVA) was applied to the analyzed chemical compounds of tomato genotypes: carbohydrates (considered as glucose + fructose), organic acids (considered as malic acid + citric acid), and total phenolic compounds (caffeic-glucoside I, caffeic-glucoside II, 3-caffeoyl quinic, 5-caffeoyl quinic, caffeic, ferulic-glucoside, Q-tri glucoside, Q-rutinoside, naringenin, ferulic). The data obtained after determining the volatile profile of tomato genotypes were subjected to multivariate statistical analysis, namely principal component analysis (PCA), using Unscrambler 11 software (Camo Analytics, Bedford, MA, USA). The statistical analysis of the data obtained from the tastings was performed by ANOVA without replication. The Duncan test (alpha < 0.05) was used as a posthoc test for the analysis of differences between tasting averages. The average values by types of tasters (women-men), depending on age categories (five groups) and tomatoes genotypes (8) were analyzed using *t*-test (Student). The phenotypic correlation among the pairs of the studied traits was analyzed by calculating Pearson coefficients of correlation, using Past software [88]. Past software was used also for CA (correspondence analysis) and PCA (a multivariate principal components analysis) graph, which was performed using the two newly commercial hybrids and their four parental lines and the chemical compounds analyzed in this study.

For the analyzed characteristics heterosis of the commercial hybrids AS 300 F1 and AS 400 F1 were calculated. The calculation of heterosis was performed as follows [89,90,91]:

- Absolute heterosis, using two ways:
  - comparing the trait (average value) of F1 hybrids with the average of the parents, using the formula:
    (1) $$H=F1-\frac{\left(P1+P2\right)}{2}$$
  - comparing the trait (average value) of F1 hybrids with the average (trait value) of the best parent, using the formula:
    H = F1 − Pmax(2)
  - Relative heterosis, or mid-parents heterosis (%) using the average values of F1 hybrids and genitors (parents), according to the formula:
    (3) $$H=\frac{F1-M.P.}{M.P.}\times 100$$
    where F1 = mean value of the trait in F1; M.P. = mean value of the two parents, as (P1 + P2)/2.
  - Heterobeltiosis (BH), as a percentage, using the average of the F1 hybrids and the best parent (Pmax) for the analyzed trait, according to the formula:
    (4) $$\mathrm{BH}=\frac{\left(F1-\mathrm{PMax}\right)}{\mathrm{PMax}}\times 100$$

Based on heterosis values, major genetic effects involved in the heterosis can be highlighted [89,90,91].

## 5. Conclusions

From the results of this study, it is concluded that even if all genotypes of the tomatoes were examined under the same conditions, the results of the chemical analysis of important components of fruits indicate significantly different values depending on the genetic differences of the improved hybrids and their analyzed parental lines. The differences in composition were manifested both between unrelated genotypes (new commercial hybrids and control ones), but also between closely related ones (parental lines and the descendant commercial hybrids resulting from their crosses). The results obtained for carbohydrates and organic acids indicate that for hybrid AS 300 F1, higher values were observed in the parental lines than in the hybrid, while in the hybrid AS 400 F1 the parental lines registered lower values than the hybrid. For phenolic compounds, in the hybrid AS 300 F1 the lines had lower values, and in the hybrid AS 400 F1 the parental lines had higher values. Regarding volatile compounds, the hybrid AS 300 F1 showed higher values than the parental lines for hexanal concentration, and in the hybrid AS 400 F1 had values almost equal to the parental lines compared to the concentration in 3-pentanones. The chemical differences also resulted in differences in the organoleptic appreciation of the fruit quality by the tasters. The consumers discriminated the taste quality and flavor depending on tomato genotypes. However, the differences in appreciation by tasting were mostly not significant between women and men, although some differences among genders were observed. In this way, women tasters gave higher marks for tomatoes’ taste compared to men if the fruits were richer in glucose (and carbohydrates, in general), and in men, the appreciation of the tomato flavor by notes was inversely proportional to the 3-Caffeoyl quinic content in the fruits. Heterosis exhibited negative values for the majority of chemical compounds. In addition to the general phenomena, also valid for tomatoes, that explain heterosis (including dominance, overdominance, and even pseudo-overdominance), the influence being determined by a different degree of homozygosity of the parental lines is also possible. Overall, the results obtained provide useful information for tomato breeding and increasing the efficiency of selection works.

## Figures and Tables

**Table 1:** Amount of carbohydrates and organic acids for tomato samples, expressed in mg/g fresh weigh.

No.	Tomato Genotype	Glucose		Fructose		Glucose + Fructose		Malic Acid		Citric Acid		Malic Acid + Citric Acid	
		Mean ± SD	Signif.	Mean ± SD	Signif.	Mean ± SD	Signif.	Mean ± SD	Signif.	Mean ± SD	Signif.	Mean ± SD	Signif.
1	Precos F1	7.17 ± 0.55	b	9.91 ± 0.60	b	17.08 ± 1.15	b	0.61 ± 0.10	e	2.40 ± 0.04	f	3.01 ± 0.14	c
2	AS 30 ♀ (F7)	7.57 ± 0.42	b	10.09 ± 0.37	b	17.66 ± 0.80	b	1.53 ± 0.06	b	2.91 ± 0.03	d	4.44 ± 0.09	b
3	AS 31 ♂ (F7)	7.59 ± 0.46	b	11.17 ± 0.76	b	18.76 ± 1.22	b	1.35 ± 0.04	c	3.00 ± 0.05	c	4.35 ± 0.01	b
4	AS 300 F1	5.75 ± 0.54	c	7.63 ± 0.85	c	13.38 ± 1.39	c	0.94 ± 0.07	d	1.73 ± 0.07	g	2.83 ± 0.21	c
5	Addalyn F1	12.15 ± 0.57	a	14.67± 0.62	a	26.82 ± 1.18	a	1.73 ± 0.09	a	3.33 ± 0.08	b	5.06 ± 0.17	a
6	AS 09 ♀ (F7)	11.38 ± 0.85	a	15.71 ± 1.02	a	27.09 ± 1.87	a	1.53 ± 0.05	b	2.72 ± 0.01	e	4.24 ± 0.04	b
7	AS 10 ♂ (F7)	12.52 ± 0.78	a	15.30 ± 0.54	a	27.82 ± 0.23	a	1.52 ± 0.09	b	3.39 ± 0.03	a	4.90 ± 0.12	a
8	AS 400 F1	11.37 ± 0.81	a	15.11 ± 0.93	a	26.47 ± 1.74	a	1.63 ± 0.05	ab	2.70 ± 0.03	e	4.33 ± 0.09	b

Values are expressed as mean ± SD. Any two means followed by the same letter are not significantly different (Duncan test, α < 0.05).

**Table 2:** Total phenolic compounds content analysed, expressed in µg/g fresh weight.

No.	Tomato Genotype	Caffeic-Glucoside I		Caffeic-Glucoside II		3-Caffeoyl Quinic Acid		5-Caffeoyl Quinic Acid		Caffeic Acid		Ferulic-Glucoside		Q-Tri Glucoside		Q-Rutinoside		Naringenin		Ferulic Acid		Total Phenolic Compounds	
		Mean ± SD	Signif.	Mean ± SD	Signif.	Mean ± SD	Signif.	Mean ± SD	Signif.	Mean ± SD	Signif.	Mean ± SD	Signif.	Mean ± SD	Signif.	Mean ± SD	Signif.	Mean ± SD	Signif.	Mean ± SD	Signif.	Mean ± SD	Signif.
1	Precos F1	6.8 ± 0.5	d	4.0 ± 0.2	e	6.5 ± 0.5	cde	6.9 ± 0.5	de	3.0 ± 0.4	c	6.1 ± 0.2	a	3.6 ± 0.4	de	2.6 ± 0.4	f	1.6 ± 0.1	d	6.8 ± 0.5	c	47.8 ± 2.6	de
2	AS 30 ♀ (F7)	2.5 ± 0.3	f	3.3 ± 0.6	e	5.7 ± 0.1	de	6.4 ± 0.5	e	2.6 ± 0.3	c	2.4 ± 0.4	e	3.1 ± 0.4	e	3.4 ± 0.6	e	2.5 ± 0.6	c	6.7 ± 0.4	c	38.4 ± 4.1	f
3	AS 31 ♂ (F7)	3.2 ± 0.6	f	3.5 ± 0.2	e	5.4 ± 0.6	e	7.2 ± 0.4	de	3.1 ± 0.4	c	3.8 ± 0.6	cd	3.8 ± 0.4	cde	4.1 ± 0.8	d	2.1 ± 0.3	cd	6.8 ± 0.8	c	42.8 ± 3.5	ef
4	AS 300 F1	4.7 ± 0.9	e	5.0 ± 0.6	d	6.8 ± 0.6	cd	7.9 ± 0.6	d	4.2 ± 0.5	b	4.2 ± 0.5	bc	3.9 ± 0.6	cd	3.7 ± 0.6	e	2.2 ± 0.6	cd	8.0 ± 0.6	b	50.3 ± 6.1	de
5	Addalyn F1	5.6 ± 0.6	e	7.4 ± 0.6	c	7.8 ± 0.6	c	12.0 ± 0.8	c	4.4 ± 0.3	b	3.2 ± 0.4	d	3.7 ± 0.3	cde	3.6 ± 0.3	e	2.3 ± 0.1	c	3.6 ± 0.4	e	53.4 ± 3.0	d
6	AS 09 ♀ (F7)	16.8 ± 0.6	a	10.2 ± 0.7	a	12.5 ± 0.6	b	16.2 ± 0.9	ab	6.0 ± 0.4	a	5.5 ± 0.4	a	7.8 ± 0.1	a	6.5 ± 0.4	c	4.8 ± 0.4	a	10.1 ± 0.7	a	96.3 ± 5.3	a
7	AS 10 ♂ (F7)	9.8 ± 0.8	b	9.0 ± 0.5	b	14.3 ± 0.6	a	17.1 ± 0.6	a	5.8 ± 0.4	a	4.6 ± 0.4	b	6.8 ± 0.5	b	10.2 ± 0.4	a	5.1 ± 0.5	a	6.0 ± 0.8	c	88.4 ± 4.5	b
8	AS 400 F1	8.5 ± 0.5	c	7.4 ± 0.1	bc	13.3 ± 0.9	ab	15.0 ± 0.5	b	5.5 ± 0.5	a	3.8 ± 0.6	cd	4.5 ± 0.5	c	7.6 ± 0.4	b	3.6 ± 0.4	b	4.6 ± 0.4	d	73.4 ± 3.8	c

Values are expressed as mean ± SD. Any two means followed by the same letter are not significantly different (Duncan test, α < 0.05).

**Table 3:** Phenotypic correlations between the pairs of characteristics analyzed (below the diagonal) and *p* value of the correlation (above the diagonal).

r.	Traits (Chemical Component)	Coefficient of Correlation (‘r’ Calculated Value between Pair of Traits; 1–17 Traits)/‘*p*’ Value																
		1	2	3	4	5	6	7	8	9	10	11	12	13	14	15	16	17
1	Glucose		0.000	0.000	0.037	0.044	0.022	0.119	0.685	0.379	0.167	0.708	0.886	0.340	0.132	0.554	0.845	0.783
2	Fructose	0.973		0.000	0.040	0.060	0.032	0.063	0.832	0.328	0.206	0.725	0.916	0.240	0.219	0.547	0.736	0.784
3	Glucose + fructose	0.992	0.994		0.037	0.050	0.025	0.083	0.761	0.348	0.183	0.715	0.992	0.280	0.172	0.548	0.784	0.783
4	Malic acid	0.738	0.729	0.738		0.050	0.002	0.640	0.428	0.753	0.393	0.277	0.359	0.331	0.782	0.915	0.823	0.945
5	Citric acid	0.719	0.687	0.707	0.707		0.001	0.852	0.836	0.832	0.734	0.116	0.855	0.360	0.770	0.348	0.743	0.241
6	Malic acid + citric acid	0.781	0.751	0.770	0.908	0.937		0.767	0.591	0.977	0.531	0.142	0.745	0.331	0.774	0.642	0.917	0.551
7	Caffeic-glucoside I	0.597	0.681	0.647	0.197	0.080	0.125		0.567	0.506	0.082	0.721	0.705	0.195	0.262	0.953	0.381	0.430
8	Caffeic-glucoside II	0.172	0.090	0.129	0.327	0.088	0.225	0.240		0.230	0.032	0.203	0.422	0.188	0.714	0.491	0.993	0.188
9	3-Caffeoyl quinic	0.361	0.398	0.384	0.133	−0.090	0.012	0.277	−0.479		0.836	0.112	0.224	0.512	0.635	0.646	0.855	0.958
10	5-Caffeoyl quinic	0.541	0.501	0.523	0.352	0.144	0.262	0.649	0.749	0.088		0.511	0.436	0.086	0.215	0.779	0.841	0.392
11	Caffeic	−0.158	−0.149	−0.154	−0.439	−0.600	−0.568	0.151	−0.504	0.604	−0.274		0.575	0.045	0.823	0.390	0.724	0.403
12	Ferulic-glucoside	−0.061	0.045	−0.004	0.376	−0.078	0.138	−0.160	−0.332	0.484	−0.323	0.235		0.555	0.151	0.417	0.888	0.565
13	Q-tri glucoside	−0.390	−0.470	−0.436	−0.396	−0.375	−0.397	−0.512	−0.518	0.274	−0.643	0.719	0.247		0.605	0.388	0.919	0.792
14	Q-rutinoside	−0.580	−0.489	−0.535	−0.118	−0.124	−0.122	−0.451	−0.155	−0.200	−0.493	−0.095	0.557	0.217		0.439	0.274	0.648
15	Naringenin	−0.248	−0.252	−0.252	0.045	−0.384	−0.196	0.025	0.287	−0.194	−0.119	0.354	0.335	0.355	0.321		0.668	0.011
16	Ferulic	0.083	0.143	0.116	−0.095	0.139	0.045	0.360	0.003	0.077	0.085	0.149	0.060	0.043	0.441	0.181		0.403
17	Total phenolic compounds	−0.117	−0.116	−0.117	0.029	−0.469	−0.250	0.326	0.519	0.022	0.352	0.345	0.241	0.112	0.193	0.829	0.345	

Correlation is significant at the level of *p* < 0.05; 0.01; 0.001 (2-tailed).

## Supplementary Materials

The following are available online at https://www.mdpi.com/article/10.3390/plants10112480/s1.
