# Supplementary material for: Physico-Chemical, Nutritional, and Sensory Evaluation of Two New Commercial Tomato Hybrids and Their Parental Lines
Source: Plants (Basel). 2021 Nov 16;10(11):2480. doi: 10.3390/plants10112480 (PMC8620249; doi:10.3390/plants10112480)
Supplement: Supplementary file 1 [file plants-10-02480-s001.zip › plants-1447263-supplementary.pdf]

SUPPLEMENTARY MATERIALS

Physico-Chemical, Nutritional, and Sensory Evaluation of Two New Commercial Tomato Hybrids and Their Parental Lines

Zoltán Felföldi [1,2], Floricuta Ranga [3], Sonia Ancuta Socaci [4], Anca Farcas [4], Mariola Plazas [5], Adriana F. Sestras [1], Dan Cristian Vodnar [3], Jaime Prohens [5] and Radu E. Sestras [1*]

[1] Faculty of Horticulture, University of Agricultural Sciences and Veterinary Medicine of Cluj-Napoca, 3-5 Manastur St., 400372 Cluj-Napoca, Romania; zoltan.felfoldi@usamvcluj.ro (Z.F.); adriana.sestras@usamvcluj.ro (A.F.S.)
[2] Private Research Station Agrosel, 268 Laminoriștilor St., 400500 Câmpia Turzii, Romania
[3] Department of Food Science and Technology, University of Agricultural Sciences and Veterinary Medicine of Cluj-Napoca, 3-5 Manastur St., 400372 Cluj-Napoca, Romania; floricuta.ranga@usamvcluj.ro (F.R.); dan.vodnar@usamvcluj.ro (D.C.V.)
[4] Department of Chemistry and Biochemistry, University of Agricultural Sciences and Veterinary Medicine of Cluj-Napoca, 3-5 Manastur St., 400372 Cluj-Napoca, Romania; sonia.socaci@usamvcluj.ro (S.A.S.); anca.farcas@usamvcluj.ro (A.F.)
[5] Institute for Conservation and Improvement of Valencian Agrodiversity (COMAV), Universitat Politècnica de València, Camí de Vera 14, 46022 Valencia, Spain; maplaav@btc.upv.es (M.P.); jprohens@btc.upv.es (J.P.);
* Correspondence: rsestras@usamvcluj.ro (R.E.S.)

Table S1. Mean relative concentrations (expressed as % of total peak areas) and standard deviations (SD) of volatile compounds from tomato varieties analysed by headspace ITEX/GC-MS technique

No.	Compound name	Genotype / Mean ±SD							
		Precos F1	AS 300 F1	AS 30 ♀ (F7)	AS 31 ♂ (F7)	Addalyn F1	AS 400 F1	AS 09 ♀ (F7)	AS 10 ♂ (F7)
1	1-Penten-3-ol							4.10 ± 0.38	10.01 ± 0.53
2	3-Pentanone					6.51 ± 0.44	8.20 ± 0.80	4.37 ± 0.42	7.32 ± 0.23
3	Pentanal	3.78 ± 0.16	5.11 ± 0.72	6.64 ± 0.36	4.81 ± 0.52				
4	n-Propyl acetate				1.19 ± 0.65				
5	1-Butanol, 3-methyl-	2.13 ± 0.01	2.48 ± 0.35	0.95 ± 0.14	8.67 ± 0.69			20.96 ± 0.56	
6	1-Butanol, 2-methyl-	1.43 ± 0.14	1.23 ± 0.38	1.61 ± 0.10	2.07 ± 0.05			4.93 ± 0.45	
7	Pentanal, 2-methyl-	0.18 ± 0.33			0.45 ± 0.08				
8	Propanoic acid, 2-methyl-, ethyl ester	0.74 ± 0.16		0.35 ± 0.14	0.45 ± 0.14				
9	1-Pentanol	0.68 ± 0.16		1.35 ± 0.37	1.77 ± 0.15	1.70 ± 0.30		1.41 ± 0.63	
10	Acetic acid, 2-methylpropyl ester				1.27 ± 0.10			0.45 ± 0.15	
11	Butanoic acid, 3-methyl-, methyl ester								
12	Hexanal	29.85 ± 1.28	24.11 ± 0.86	12.97 ± 0.41	30.02 ± 0.41	8.58 ± 0.87	4.11 ± 0.47	0.83 ± 0.33	1.41 ± 0.36
13	Furfural								1.66 ± 0.34
14	Butanoic acid, 3-methyl-, ethyl ester		0.90 ± 0.17	0.19 ± 0.18					
15	1-Hexanol	0.25 ± 0.20		1.08 ± 0.14	1.61 ± 0.12	2.54 ± 0.78	1.30 ± 0.58	1.62 ± 0.36	2.14 ± 0.60
16	1-Butanol, 3-methyl-, acetate				4.51 ± 0.58			17.68 ± 1.42	
17	1-Butanol, 2-methyl-, acetate				0.65 ± 0.33			1.17 ± 0.67	
18	Heptanal	2.08 ± 0.07	0.75 ± 0.26	1.06 ± 0.62	1.76 ± 0.21				
19	1-Butanol, 2-ethyl-						1.54 ± 0.42	5.11 ± 0.73	2.90 ± 0.35
20	Acetic acid, pentyl ester				0.19 ± 0.40			1.06 ± 0.36	
21	Bicyclo[3.1.0]hex-2-ene, 2-methyl-5-(1-methylethyl)- *	0.34 ± 0.22			0.23 ± 0.45				
22	Hexanoic acid, methyl ester	0.12 ± 0.14		0.22 ± 0.23	0.07 ± 0.19				
23	.alpha.-Pinene	0.85 ± 0.19		0.15 ± 0.14	0.26 ± 0.36				
24	Benzene, 1-methoxy-4-methyl- *			0.75 ± 0.34	0.12 ± 0.30	0.92 ± 0.55			
25	2-Heptanone, 6-methyl-	0.33 ± 0.40		0.28 ± 0.33		0.20 ± 0.04	0.28 ± 0.58		
26	2-Heptenal, (Z)-	0.80 ± 0.14	1.13 ± 0.45	0.71 ± 0.17	0.84 ± 0.20	0.36 ± 0.02			
27	Benzaldehyde	1.34 ± 0.30	2.76 ± 0.15	2.70 ± 0.14	0.77 ± 0.48	2.45 ± 0.33	2.84 ± 0.43	1.11 ± 0.31	2.99 ± 0.38

28	Bicyclo[3.1.0]hexane, 4-methylene-1-(1-methylethyl)-*	1.20 ± 0.16		0.20 ± 0.14	0.19 ± 0.32				
29	Heptanol							0.49 ± 0.10	
30	.beta.-Pinene	3.34 ± 0.55		0.92 ± 0.29	0.64 ± 0.34	0.27 ± 0.12			
31	1-Octen-3-one	0.25 ± 0.28			0.26 ± 0.36				
32	Phenol	1.19 ± 0.03	2.09 ± 0.04	2.58 ± 0.13	0.71 ± 0.16	2.47 ± 0.21	1.72 ± 0.49	1.12 ± 0.38	2.25 ± 0.34
33	5-Hepten-2-one, 6-methyl-	4.82 ± 0.42	5.62 ± 0.25	11.88 ± 0.40	6.13 ± 0.70	10.81 ± 1.11	19.52 ± 0.73	6.80 ± 0.28	19.72 ± 0.74
34	.beta.-Myrcene	10.59 ± 0.50							
35	Furan, 2-pentyl-		15.03 ± 1.07	13.99 ± 0.62	8.83 ± 0.42	19.59 ± 1.59	10.07 ± 0.69	7.28 ± 0.43	16.06 ± 0.43
36	5-Hepten-2-ol, 6-methyl-			0.28 ± 0.33	0.08 ± 0.40	0.15 ± 0.08			
37	trans-2-(2-Pentenyl)furan *	0.28 ± 0.42		1.65 ± 0.21	0.16 ± 0.20	0.25 ± 0.36	0.52 ± 0.24	0.23 ± 0.46	0.35 ± 0.07
38	Hexanoic acid, ethyl ester	0.24 ± 0.22	0.28 ± 0.19		0.53 ± 0.14				
39	Octanal	2.79 ± 0.24	0.75 ± 0.14		1.15 ± 0.16	1.25 ± 0.29		0.37 ± 0.26	0.86 ± 0.21
40	3-Carene	0.41 ± 0.18					1.32 ± 0.16		
41	Acetic acid, hexyl ester				0.12 ± 0.13			0.83 ± 0.24	0.44 ± 0.06
42	.alpha.-Terpinene	0.29 ± 0.09							
43	p-Cymene	10.1 ± 0.45	1.74 ± 0.26	6.22 ± 0.63	4.64 ± 0.14	7.39 ± 0.67	0.99 ± 0.32	2.50 ± 0.44	0.23 ± 0.08
44	1,3,7-Octatriene, 3,7-dimethyl- *	0.13 ± 0.01							
45	.gamma.-Terpinene	5.12 ± 0.55		3.55 ± 0.14		4.16 ± 0.50		1.19 ± 0.67	
46	2-Octenal, (E)-		4.31 ± 0.57		2.13 ± 0.48		1.26 ± 0.43		0.69 ± 0.24
47	Acetophenone	1.22 ± 0.15	2.40 ± 0.21	2.27 ± 0.19	0.70 ± 0.14	2.38 ± 0.19	2.97 ± 0.33	0.58 ± 0.14	2.33 ± 0.15
48	2-Octen-1-ol					0.99 ± 0.46			
49	1-Octanol	0.29 ± 0.11		1.72 ± 0.28	0.70 ± 0.14	0.77 ± 0.14	1.05 ± 0.07	2.06 ± 0.35	2.18 ± 0.16
50	.alpha.- Terpinolene	0.32 ± 0.12							
51	Furan, 3-(4-methyl-3-pentenyl)-			1.28 ± 0.35		1.06 ± 0.19	3.70 ± 0.31	1.02 ± 0.40	3.06 ± 0.36
52	Nonanal	1.13 ± 0.24	0.64 ± 0.14	1.11 ± 0.31	0.89 ± 0.15	1.07 ± 0.42	1.69 ± 0.45	0.35 ± 0.06	0.94 ± 0.14
53	Octanoic acid, methyl ester	0.38 ± 0.12	0.41 ± 0.16	0.28 ± 0.29	0.18 ± 0.33	0.35 ± 0.62			
54	2-Nonenal, (E)-			5.24 ± 0.45	0.85 ± 0.25				
55	Benzoic Acid		16.08 ± 0.73	3.05 ± 0.35	2.09 ± 0.08	6.60 ± 0.8	23.25 ± 0.84	6.13 ± 0.10	4.12 ± 0.45
56	Octanoic Acid			0.48 ± 0.45	1.00 ± 0.46				
57	1-Nonanol								
58	2-Dodecanone*	0.15 ± 0.12		0.18 ± 0.38	0.08 ± 0.09		0.13 ± 0.29		
59	Methyl Salicylate					3.32 ± 0.39			3.01 ± 0.38
60	Octanoic acid, ethyl ester	0.57 ± 0.13	0.63 ± 0.09		2.00 ± 0.31				
61	Decanal	0.61 ± 0.12	0.62 ± 0.25	0.81 ± 0.29	0.24 ± 0.22	0.78 ± 0.15	0.88 ± 0.16	0.35 ± 0.07	0.24 ± 0.22
62	Acetic acid, octyl ester							0.78 ± 0.16	
63	5-Hydrxoymethylfurfural	0.58 ± 0.18	2.97 ± 0.19						2.98 ± 0.48

64	Nonanoic acid	0.26 ± 0.05				0.29 ± 0.12			
65	2,6-Octadienal, 3,7-dimethyl-, (E)- *	0.24 ± 0.03	0.24 ± 0.17	0.57 ± 0.40	0.18 ± 0.19	0.26 ± 0.10	0.66 ± 0.07		0.35 ± 0.06
66	Benzoic acid, 2-hydroxy-, ethyl ester		0.77 ± 0.26						
67	Ethanone, 1-[4-(1-meth-ylethyl)phenyl]-								
68	Undecanal	0.16 ± 0.11							
69	2-Undecenal	0.19 ± 0.06			0.38 ± 0.19				
70	Dodecanal	0.17 ± 0.57							
71	5,9-Undecadien-2-one, 6,10-di-methyl-, (Z)-	1.85 ± 0.19	3.22 ± 0.47	3.45 ± 0.48	1.49 ± 0.33	3.61 ± 0.45	4.10 ± 0.46	2.12 ± 0.16	4.51 ± 0.26
72	1-Dodecanol	1.90 ± 0.31	0.65 ± 0.35						
73	Dodecanoic acid	4.32 ± 0.75	3.10 ± 0.56	7.29 ± 0.60	1.92 ± 0.50	8.94 ± 1.2	7.90 ±0.38	2.14 ± 0.45	7.25 ± 0.43

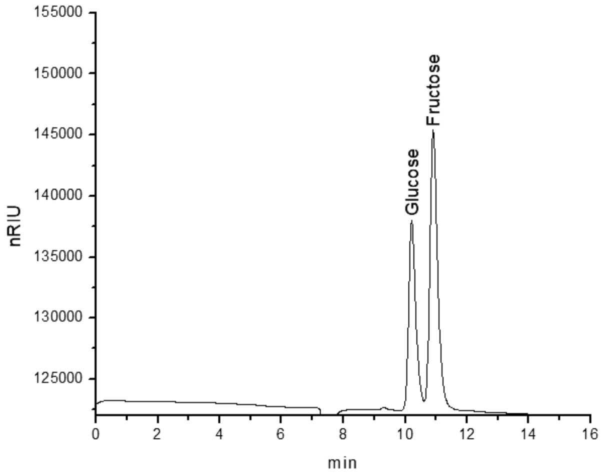

Figure S1. Chromatogram of carbohydrate standards and retention time: glucose R_t = 10.22 min; fructose R_t = 10.87 min

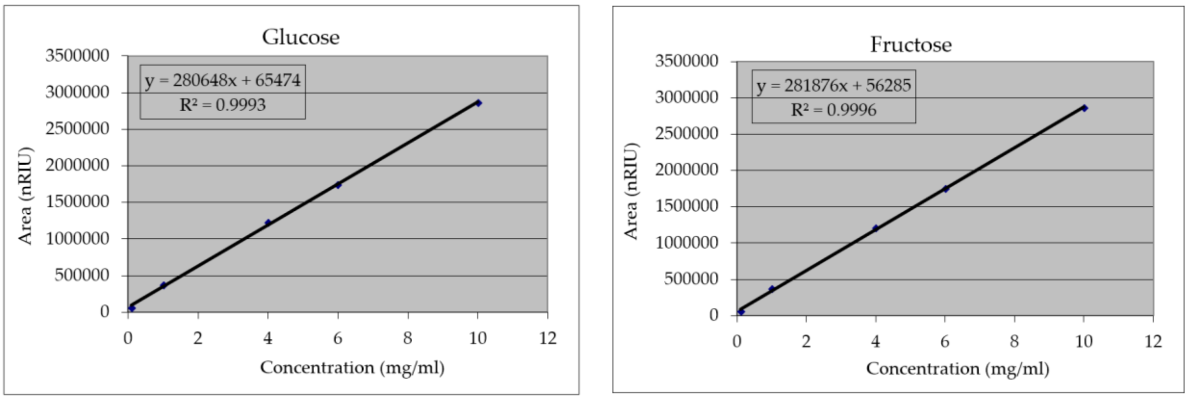

(**a**) (**b**)

Figure S2. Calibration curves with glucose and fructose standards of 99% purity, with mg/ml concentration: (**a**) fructose R^2 = 0.9996; (**b**) glucose R^2 = 0.9993; $P < 0.05$)

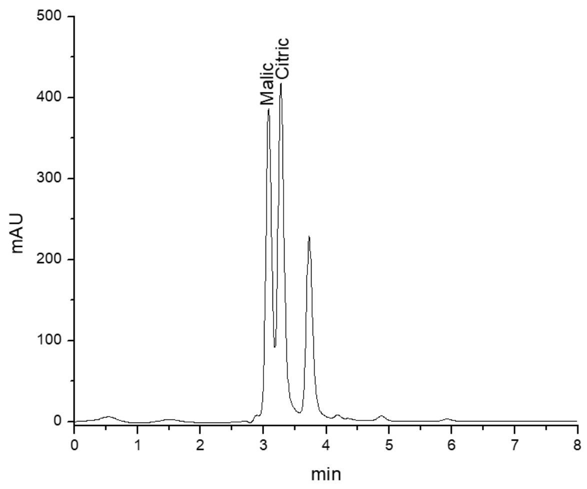

Figure S3. Chromatogram of organic acid standards and retention time: malic acid Rt = 3.08 min, citric acid Rt = 3.32 min

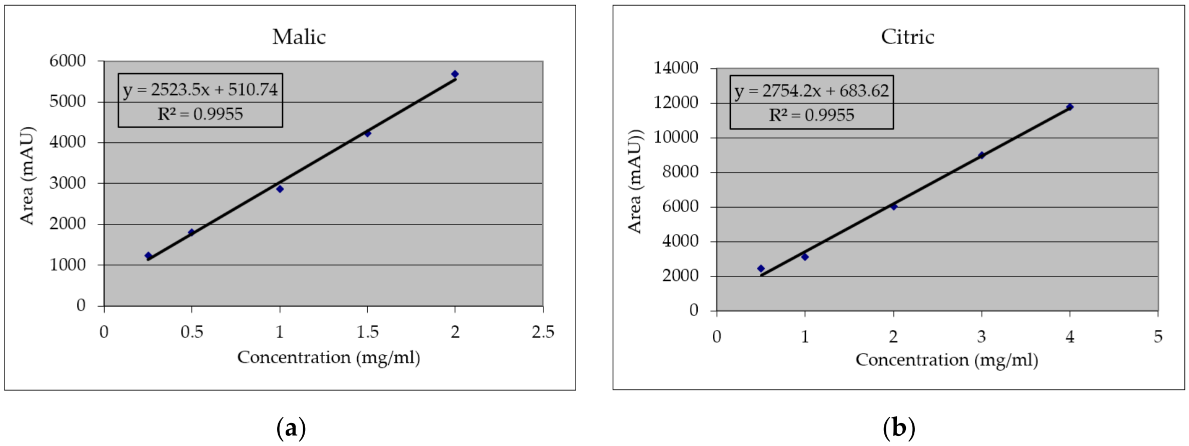

Figure S4. Calibration curves for organic acids by injecting five different concentrations of standard substance of 99% purity: (**a**) Malic acid $R^2 = 0.9955$; (**b**) Citric acid $R^2 = 0.9955$)

Table S2. Identification of phenolic compounds performed after retention times, UV-Vis and mass spectra for each peak.

Peak	Rt (min)	λmax (nm)	[M+H] + (m/z)	Phenolic compounds	Phenolic subclasses
1	10.87	302, 245	343	Caffeic acid-glucoside I	Hidroxycinamic acid
2	11.85	312, 248	343	Caffeic acid-glucoside I	Hidroxycinamic acid
3	12.29	326, 248	355	3-Caffeoylquinic acid	Hidroxycinamic acid
4	12.48	326, 248	355	5-Caffeoylquinic acid	Hidroxycinamic acid
5	13.64	324, 248	181	Caffeic acid	Hidroxycinamic acid
6	14.03	328, 248	357	Ferulic acid-glucoside	Hidroxycinamic
7	14.68	355, 255	743	Quercetin-triglucoside	Flavonol
8	15.69	354, 256	611	Quercetin-rutinoside	Flavonol
9	16.03	330, 280	580	Naringin	Flavanone
10	17.32	326, 248	195	Ferulic acid	Hidroxycinamic

*Spectral values were recorded in the range 200-600 nm for all peaks, and chromatograms were recorded at the wavelength λ = 340 nm. For the retention time (Rt) values were recorded between 10.87-17.32 min, and the mass spectrum $[M + H]^+$ had values between 181-743 m/z.

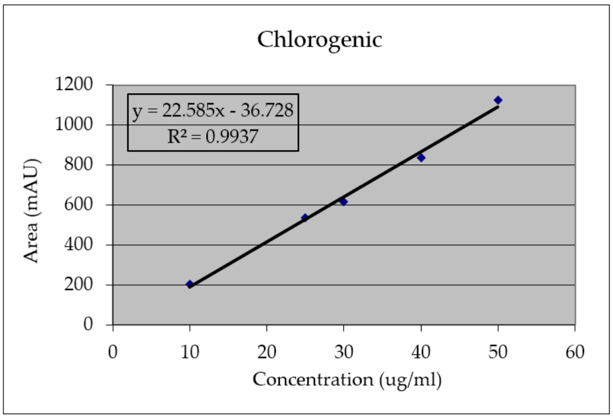

(**a**)

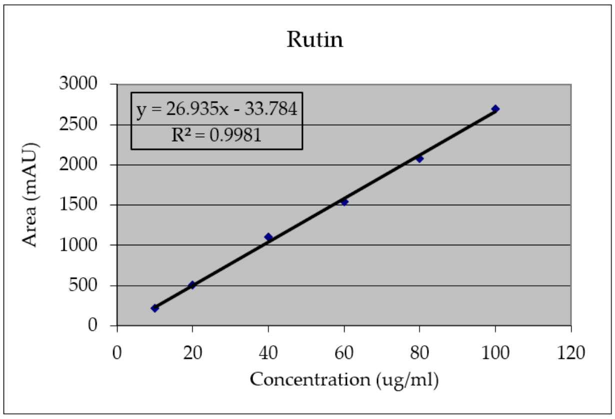

(**b**)

Figure S5. Calibration curves for total phenolic content with chlorogenic and rutin standard of 99% purity, with ug/ml concentration: (**a**) Chlorogenic R^2=0.9937; (**b**) Rutin R^2=0.9981, P<0,05)
